# Link prediction on Twitter

**DOI:** 10.1371/journal.pone.0181079

**Published:** 2017-07-18

**Authors:** Sanda Martinčić-Ipšić, Edvin Močibob, Matjaž Perc

**Affiliations:** 1 Department of Informatics, University of Rijeka, Rijeka, Croatia; 2 Faculty of Natural Sciences and Mathematics, University of Maribor, Maribor, Slovenia; 3 Center for Applied Mathematics and Theoretical Physics, University of Maribor, Maribor, Slovenia; University of Warwick, UNITED KINGDOM

## Abstract

With over 300 million active users, Twitter is among the largest online news and social networking services in existence today. Open access to information on Twitter makes it a valuable source of data for research on social interactions, sentiment analysis, content diffusion, link prediction, and the dynamics behind human collective behaviour in general. Here we use Twitter data to construct co-occurrence language networks based on hashtags and based on all the words in tweets, and we use these networks to study link prediction by means of different methods and evaluation metrics. In addition to using five known methods, we propose two effective weighted similarity measures, and we compare the obtained outcomes in dependence on the selected semantic context of topics on Twitter. We find that hashtag networks yield to a large degree equal results as all-word networks, thus supporting the claim that hashtags alone robustly capture the semantic context of tweets, and as such are useful and suitable for studying the content and categorization. We also introduce ranking diagrams as an efficient tool for the comparison of the performance of different link prediction algorithms across multiple datasets. Our research indicates that successful link prediction algorithms work well in correctly foretelling highly probable links even if the information about a network structure is incomplete, and they do so even if the semantic context is rationalized to hashtags.

## Introduction

Our cumulative culture relies on our ability to carry the knowledge from previous generations forward. For millennia, we have been upholding a cumulative culture, which leads to an exponential increase in our cultural output [1], and it has given us evolutionary advantages that no other species on the planet can compete with. Unprecedented technological progress and scientific breakthroughs today make the amount of information to carry forward staggering. This requires information sharing, worldwide collaboration, the algorithmic prowess of search engines, as well as the selfless efforts of countless volunteers to maintain, categorize, and help navigate what we know. The task is made easier by the fact that much of what we know has been digitized [2, 3]. The combination of data deluge with recent advances in the theory and modeling of social systems and networks [4–12] enables quantitative explorations of our culture that were unimaginable even a decade ago. Recent research has been devoted to enhanced disease surveillance [13], the spreading of misinformation [14, 15], to study human mobility patterns [16, 17] and the dynamics of online popularity [18], to quantify trading behavior [19, 20] and the dynamics of our economic life [21], as well as to study universality in voting behavior [22], political polarity [23] and emotional blogging [24, 25], to name just some examples.

The openness of Twitter to research has made it an important source of data for innovative data-driven research that lifts the veil on how we share information, how and with whom we communicate, and essentially on how we live our lives. Twitter was created in 2006, enabling users to send short publicly visible messages called tweets. Tweets typically consist of text, links (i.e. URLs), user mentions (with @ sign), retweet information (RT) and hashtags. Hashtags are marked with the # sign and are used for meta tagging, which enables users to find a specific theme or content [26]. Hashtags are neither limited nor do they have a predefined structure or content. Still they often capture the very essence of posted messages, much like keywords or keyphrases do [27], and they can be used effectively to monitor trends of topics on Twitter [28] as well as the polarity of tweets [29]. So far, Twitter data has been used to study the growth mechanisms of social interactions [30], for assessing user influence [31], for recommending (predicting) whom to follow [32], for information propagation [33], as well as for sentiment analysis [29, 34, 35].

Here we use Twitter data to study link prediction in the realm of co-occurrence language networks based on hashtags and based on all the words in tweets. Link prediction refers to inferring the future relationships from nodes in the complex network, or more formally, to estimate the likelihood of the existence of a link between two nodes based on the observed network structure and node attributes. A comprehensive review of link prediction methods is provided in [36]. In addition to relying on topological properties of networks, the problem was also addressed by the means of various machine learning techniques [37, 38]. Typical networks addressed by means of link prediction methods include protein-protein interaction networks and social networks, where one can predict longitudinal changes over time [36, 39–42]. While local similarity measures have traditionally been explored for unweighted networks, recently weighted local similarity measures have attracted more attention [37, 43–46]. In line with these trends, we therefore focus on weighted local similarity measures for the prediction of links in the networks constructed from the content of tweets.

In addition to using five known methods, namely the weighted common neighbors (CN), the weighted Jaccard coefficient (JC), the weighted preferential attachment (PA), the weighted Adamic-Adar (AA) and the weighted resource allocation index (RA) [37, 44, 47], we also propose selectivity (SE) [48] and inverse selectivity (IS) as two effective weighted similarity measures. Selectivity is defined as the average weight distributed on the links incident to the single node, and has proven efficient for different language network tasks, ranging from the differentiation between original and shuffled text [49] to the differentiation of text genres [50] and for keyword extraction [51, 52]. We also note that link prediction on Twitter has been studied before in [53], where CN, AA, JC and RA measures were combined with the information about corresponding communities as determined with a variant of the label propagation algorithm in unweighted and directed networks. It was shown that this leads to an improvement of the area under the receiver operating characteristic curve (AUC) when structural measures are accompanied with community information to train supervised data mining models for link prediction. In [41] an approach has been proposed to predict future links in Twitter reciprocal reply networks by applying the covariance matrix adaptation evolution strategy to optimize weights based on neighbourhood and node similarity indices. It was shown that this method is suitable for predicting future followers on social networks.

As we will show after describing the Methods, our research reveals that hashtag networks yield to a large degree equal results as all-word networks, therefore supporting the claim that hashtags alone robustly capture the semantic context of tweets, and as such are useful and suitable for studying the structure of tweets. We will also show how introducing ranking diagrams is an efficient tool for the comparison of the performance of different link prediction algorithms across multiple datasets.

## Methods

The network *G* = (*V*, *E*) is a pair of a set of nodes *V* (or vertices) and a set of links *E* (or edges), where *N* is the number of nodes and *K* is the number of links. In weighted networks every link connecting two nodes *u* and *v* has an associated weight *w*_*uv*_. A node degree *deg*(*u*) is the number of links incident to node *u* and the set of neighbor nodes to a node *u* is denoted as Γ(*u*). The strength of a node *s*_*u*_ is the sum of weights of all the links incident to *u*. More details about complex networks analysis can be found in [54] and all measures used for the quantification of the studied networks properties are listed in S1 Text.

There are various approaches for the link prediction task based upon similarity measures [36, 40]. In general each pair of nodes *u* and *v* (*u*, *v* ∈ *V*) is assigned a score *p*_*uv*_ which is directly defined as the similarity between nodes *u* and *v*. Then the link prediction task is to determine whether the link between *u* and *v* will be established according to the descending order of assigned scores *p*_*uv*_. Next we define seven link prediction measures used in this study.

In the weighted common neighbors (CN) link prediction measure weights of links connecting nodes *u* and *v* to their common neighbors *z* are calculated as in [44]:

$$CN\left(u,v\right)=\sum_{z\in \Gamma (u)\cap z\in \Gamma (v)}\left(w_{uz}+w_{vz}\right)$$ (1)

where Γ(*u*) and Γ(*v*) are the sets of neighbors of nodes *u* and *v*. CN measures the number of neighbors that two nodes have in common, while for the weighted CN the sum of weights is used instead. CN is the simplest but at the same time computationally undemanding measure which serves as a baseline for link prediction.

The weighted Jaccard coefficient (JC) adapted from [37], divides the weighted common neighbors value for *u* and *v* by the sum of weights on all the links incident to *u* and/or *v*:

$$\begin{array}{c} JC\left(u,v\right)=\frac{\sum_{z\in \Gamma (u)\cap z\in \Gamma (v)}\left(w_{uz}+w_{vz}\right)}{\sum_{a\in \Gamma (u)}w_{au}+\sum_{b\in \Gamma (v)}w_{bv}}. \end{array}$$ (2)

JC has been a well established measure in the information retrieval and data mining community and quantifies the probability that a common neighbour of a pair of nodes would be selected if the selection is performed randomly from the union of sets of neighbors Γ(*u*) and Γ(*v*) [40].

The weighted preferential attachment (PA) is according to [37]:

$$\begin{array}{c} PA\left(u,v\right)=\sum_{a\in \Gamma (u)}w_{au}*\sum_{b\in \Gamma (v)}w_{bv}. \end{array}$$ (3)

PA considers only the degrees of two nodes, while weighted PA also considers their weights. It has been shown that PA governs the evolving of scale-free networks [55, 56].

The weighted Adamic-Adar (AA) adapted from [37], according to the original unweighted definition in [47], is:

$$\begin{array}{c} AA\left(u,v\right)=\sum_{z\in \Gamma (u)\cap z\in \Gamma (v)}\frac{w_{uz}+w_{vz}}{\log(1+\sum_{a\in \Gamma (z)}w_{za})}. \end{array}$$ (4)

AA ranks the common neighbors with a smaller degree more heavily, and punishes the common neighbors with a higher degree.

The weighted resource allocation index (RA) where *s*_*z*_ is the strength of node *z* is defined in [44] as:

$$\begin{array}{c} RA\left(u,v\right)=\sum_{z\in \Gamma (u)\cap z\in \Gamma (v)}\frac{w_{uz}+w_{vz}}{s_{z}}. \end{array}$$ (5)

RA punishes the common neighbors with higher strength more heavily and promotes the ones with lower strength. It assumes the amount of resources that the node can share in its neighbourhood. RA was initially defined as $\sum_{z\in \Gamma (u)\cap z\in \Gamma (v)}\frac{1}{s_{z}}$ [57]. Since Lü and Zhou [44] report that the unweighted resource allocation index sometimes performs better then the weighted, we decided to use the unweighted variant of RA. The unweighted RA is governed by the same underpinning idea as selectivity and this will allow better insights into a comparative analysis of RA with two newly proposed measures.

Selectivity (SE) is defined as

$$\begin{array}{c} SE\left(u,v\right)=\sum_{z\in \Gamma (u)\cap z\in \Gamma (v)}\frac{s_{z}}{deg(z)} \end{array}$$ (6)

where *deg*(*z*) is the degree and *s*_*z*_ is the strength of node *z*. Selectivity, originally proposed by Masucci and Rogers [48], promotes the nodes with high strength and low degree, and depresses the high degree nodes. The same governing principle is exploited in the Adamic-Adar and resource allocation index. Since resource allocation has been very successful in link prediction we were motivated to test inverse selectivity as the potential link prediction measure as well.

Inverse selectivity (IS) is defined as a degree of node *z* divided by it’s strength:

$$\begin{array}{c} IS\left(u,v\right)=\sum_{z\in \Gamma (u)\cap z\in \Gamma (v)}\frac{deg(z)}{s_{z}}. \end{array}$$ (7)

Resource allocation index, selectivity and inverse selectivity are all computationally undemanding. In order to summarize the seven link prediction measures we systematically list their notation and the corresponding equations in Table 1.

**Table 1:** Summary of link prediction measures.

Measure	Notation	Equation
Weighted common neighbors	CN	*CN*(*u*, *v*) = ∑_*z* ∈ Γ(*u*)∩*z* ∈ Γ(*v*)_(*w*_*uz*_ + *w*_*vz*_)
Weighted Jaccard coefficient	JC	$JC\left(u,v\right)=\frac{\sum_{z\in \Gamma (u)\cap z\in \Gamma (v)}\left(w_{uz}+w_{vz}\right)}{\sum_{a\in \Gamma (u)}w_{au}+\sum_{b\in \Gamma (v)}w_{bv}}$
Weighted preferential attachment	PA	*PA*(*u*, *v*) = ∑_*a* ∈ Γ(*u*)_ *w*_au_*∑_*b* ∈ Γ(*v*)_ *w*_*bv*_
Weighted Adamic-Adar	AA	$AA\left(u,v\right)=\sum_{z\in \Gamma (u)\cap z\in \Gamma (v)}\frac{w_{uz}+w_{vz}}{\log(1+\sum_{a\in \Gamma (z)}w_{za})}$
Weighted resource allocation index	RA	$RA\left(u,v\right)=\sum_{z\in \Gamma (u)\cap z\in \Gamma (v)}\frac{w_{uz}+w_{vz}}{s_{z}}$
Selectivity	SE	$SE\left(u,v\right)=\sum_{z\in \Gamma (u)\cap z\in \Gamma (v)}\frac{s_{z}}{deg(z)}$
Inverse selectivity	IS	$IS\left(u,v\right)=\sum_{z\in \Gamma (u)\cap z\in \Gamma (v)}\frac{deg(z)}{s_{z}}$

In particular, *u*, *v*, *z*, *a*, *b* are nodes, *w* are weights on the links, *s*_*u*_ is the strength, *deg*(*u*) is the degree, and Γ(*u*) is the set of neighbors of the node *u*.

### Evaluation metrics

In order to test the performance of weighted similarity measures we need to establish a testing set of links *E*_*P*_ which is used as a golden standard for evaluation. When we usually use a hold-out strategy for the construction of the test set it holds that the intersection of the training *E*_*T*_ and testing *E*_*P*_ sets is empty *E*_*T*_ ∩ *E*_*P*_ = ∅ and that *E*_*T*_ ∪ *E*_*P*_ = *E*. However, in our case we followed different principles for the construction of the testing set. The data is divided into four longitudinally growing subsets, meaning that each of the three training sets is a subset of the testing set.

The link prediction can be evaluated by many different scores as elaborated in [58]. In this work we use: precision, F1 score and the area under the receiver operating characteristic curve (AUC).

The link prediction precision *P* is the ratio between the number of correctly predicted links and the total number of predicted links—the number of true positives (|*TP*|) divided by the number of true positives and false positives (|*TP*| + |*FP*|) [58] as:

$$\begin{array}{c} P=\frac{\left|TP\right|}{\left|TP|+|FP\right|}. \end{array}$$ (8)

The F1 score is a standard measure for evaluation in information retrieval tasks and is calculated as the harmonic mean of precision *P* and recall *R*:

$$\begin{array}{c} F1=2\cdot \frac{P\cdot R}{P+R}=\frac{2\cdot |TP|}{2\cdot |TP|+|FP|+|FN|} \end{array}$$ (9)

where recall is calculated as a fraction of true positives (|*TP*|) over the number of true positives and false negatives (|*TP*| + |*FN*|).

The area under the receiver operating characteristic curve (AUC) represents the performance trade-off between the true positive rate against the false positive rate [58, 59]. The receiver operator characteristic curve connects the points corresponding to the pairs of true positive and false positive rates obtained for different decision boundaries. The true positive rate is defined as the fraction of actual positive cases over all positive cases as *correct positives/total positives* or |*TP*|/(|*TP*| + |*FN*|). The false positive rate is the fraction of actual negative cases that are misclassified as positives over all negative cases as *incorrect negatives/total negatives* or |*FP*|/(|*TN*| + |*FP*|). The AUC is calculated as the area under the receiver operating characteristic curve and has values between 0 and 1. The AUC value of 0.5 is a random prediction and higher values are achieved for better models. Hence, the value of 1 represents the score of the perfect model (classifier).

**Fig 1:**
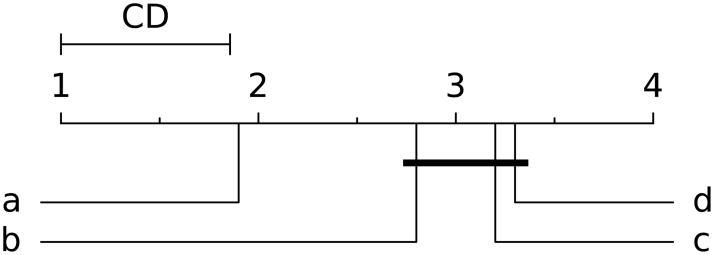
This ranking diagram shows the average ranks for 4 models (methods, classifiers): a, b, c and d. The best ranked (the best performing) model **a** is at the leftmost position, while the worst performing model **d** is ranked at the rightmost position. Others are in the middle according to the achieved rank (measured performance value). The line below shows that the difference between models **b**, **c** and **d** is not statistically significant.

The comparison of different measures for link prediction on several datasets using three evaluation metrics simultaneously amounts to the problem of comparing multiple classifiers over multiple datasets. In order to provide a better insight into the obtained results, we introduce the rank diagrams proposed by Demšar [60]. The rank diagrams position the best value on the left (1st rank) and the worst on the right side, while others are ranked in between. The groups of scores which are not significantly different are connected with the line below the x-axis. The scores (average ranks) are significantly different, if their difference is above the threshold value obtained using the Nemenyi post-hoc test: the threshold is referred to as critical distance *CD*, calculated as $CD=q_{\alpha }\sqrt{\frac{K(K+1)}{6N}}$ where *q*_*α*_ is based on Studentized range statistic, *K* is the number of models (classifiers), and *N* is the number of measurements (datasets). The critical distance value is depicted on the ranking diagram using a line above the x-axis (labeled *CD*). All rank diagrams are generated for the Nemenyi test with *p*-values below 0.05. Fig 1 shows an example of the rank diagram. The source code and the explanation of the rank diagrams is available at the Orange Data Mining webpage of the Bioinformatics Lab at the University of Ljubljana.

### Datasets

For the link prediction task we exploited two Twitter datasets: the first consists of extracted tweets using the Twitter API (referred to as emo-net) and the second consists of the Sentiment140 corpus with carefully annotated tweets according to their polarity [61] (referred to as SC).

In the emo-net corpus, we extracted four sets of tweets in the English language according to the following search criteria: a) tweets associated to immigrant and war related events (e.g. terrorist, terrorism, ISIS, etc.); b) tweets containing negatively polarized words (e.g. anger, fear, hate, etc.); c) tweets associated to pets (e.g. puppy, kitty, etc.) and d) tweets containing positively polarized words (e.g. joy, happiness, happy, etc.). We will refer to the networks constructed from these sets of tweets respectively as: a) emo-net^*a*^, b) emo-net^*b*^, c) emo-net^*c*^ and d) emo-net^*d*^. The four search criteria are selected in order to ensure consistency with the positively or negatively annotated polarity of tweets in the SC dataset, and to keep the data used for the experimental set-up comparable.

The second corpus, SC, consists of four datasets extracted from the SC’s training data as follows: a) the first 10,000 negatively polarized tweets, b) the first 10,000 positively polarized tweets, c) the first 100,000 negatively polarized tweets and d) the first 100,000 positively polarized tweets. We will refer to these datasets respectively as: a) ${\text{SC}}_{neg}^{10^{4}}$, b) ${\text{SC}}_{pos}^{10^{4}}$, c) ${\text{SC}}_{neg}^{10^{5}}$ and d) ${\text{SC}}_{pos}^{10^{5}}$. The *SC* dataset prepared in 2009 is available at http://help.sentiment140.com/for-students/.

Both corpora were subject to the same data-cleaning procedure of stopwords’ removal and tokenization at the white spaces in tweets. Table 2 summarizes the content of the eight datasets of the English tweets. It is worth noticing that the first six datasets are approximately of the same size (counted in the number of tweets). Also, $\text{S}{\text{C}}^{10^{4}}$ datasets are proper subsets of $\text{S}{\text{C}}^{10^{5}}$ datasets respectively.

**Table 2:** Eight datasets of English tweets considered in this paper.

	Number of				
Dataset	tweets	words	diff.words	hashtags	diff.hashtags
emo-net^*a*^	9987	169045	26528	7967	1592
emo-net^*b*^	9958	151216	25013	1859	985
emo-net^*c*^	9946	137291	26953	2576	1522
emo-net^*d*^	9991	143516	31983	3987	2092
${\text{SC}}_{neg}^{10^{4}}$	10000	135751	27056	185	151
${\text{SC}}_{pos}^{10^{4}}$	10000	130531	30441	183	158
${\text{SC}}_{neg}^{10^{5}}$	100000	1349841	150611	1843	1087
${\text{SC}}_{pos}^{10^{5}}$	100000	1283953	175722	2394	1324

In the emo-net dataset the tweets are extracted according to positive and negative search criteria (e.g. fear, hate, joy, puppy, etc.), while in the SC dataset tweets are selected from already annotated positive and negative polarity of the tweets [61]. The number of different words and the number of different hashtags exclude repetitions, while the number of words and hashtags are the total values including repetitions. We note that the $\text{S}{\text{C}}^{10^{4}}$ datasets are proper subsets of the larger $\text{S}{\text{C}}^{10^{5}}$ datasets.

For the data preparation we use Python in combination with the Python Twitter Tools package, which provides an easy-to-use interface for the official Twitter API. The extraction during February 2016 resulted in approximately 10,000 tweets for each of the four different datasets, constructing a corpus of 39,882 tweets in total. The raw emo-net dataset is available at http://langnet.uniri.hr/resources.html.

### Network construction

The language networks construction principle arises from the very nature of the text [48, 62, 63]. The co-occurrence relation in language networks is established between linguistic units within a sentence (here tweet), where the direction of a link reflects the words’ sequencing and weight on the link reflects the frequency of word-pairs mutual appearance—weight is the number of tweets in which two words co-occur. For the link prediction task we construct all the networks as undirected and weighted.

First we construct the networks from all the words in the tweets. From emo-net datasets we extract the top 200 most frequent words and extend the list with explicit keywords used for the extraction of tweets (e.g. joy, puppy, anger, …). A link between two nodes is established if these two words co-occur in the same tweet. For the SC datasets we retain the same principles of extracting the top 200 most frequent words and network construction. Next we construct hashtag networks. From both datasets we extract the top 200 most frequent hashtags, and a link is established between hashtags co-occurring in a tweet. Note that the number of different hashtags in ${\text{SC}}_{neg}^{10^{4}}$ and ${\text{SC}}_{pos}^{10^{4}}$ is below 200 (see values listed in Table 2), so we use the available top-frequent set. The principle of using the top 200 most frequent words (hastags) provides the best trade-off between computation time and link prediction results. Still, in order to test whether using the larger top set contributes to the change in the results we also probe the top 500 extracted hashtags in the ${\text{SC}}_{pos}^{10^{5}}$ dataset.

Finally, for each of the eight datasets for all-words and for hashtags respectively, we create subnetworks by adding 25%, 50% and 75% of the links, while the entire network of 100% links serves as the baseline for evaluation. The subnetworks preserve the temporal aspect of network construction process, since links are added according to the time of creation captured in the tweet’s timestamps. In other words, we construct networks from the sorted list of tweets (from the oldest to the newest).

To summarize, in total we construct 64 networks (32 based on all-words and 32 based on the hashtags in the tweets), systematically using 25%, 50%, 75% and 100% of the links. Network construction and analysis was implemented with the Python programming language using the NetworkX software package developed for the creation, manipulation, and study of the structure, dynamics, and functions of complex networks [64].

### Link prediction

The link prediction process is the same across all networks (25%, 50% and 75% of the links), regardless of whether the networks are constructed for the co-occurrence of all-words or hashtags in tweets. First, for each dataset we establish the test dataset *E*_*P*_ as a full network with 100% of the links. Then the link candidates are selected from all non-existing links in the current network (25%, 50% and 75%) and ranked according to the assigned value of the link prediction measures. Then we cut off the top *n* potential links, where *n* is the total number of new links in the respective testing network, and construct a candidate set. The full set of valid (true positive) future links is generated from the 100% network. Then, two sets (predicted and real links—true positive) are used for the evaluation in terms of precision, the F1 score and the area under the receiver operating characteristic curve (AUC).

## Results

In this section, we show all the results needed to communicate the main message of our research, while additional results are provided in the S1 Text, together with the definition of a standard set of network measures used for exploring the structure of networks.

### Link prediction results in all-word networks

**Fig 2:**
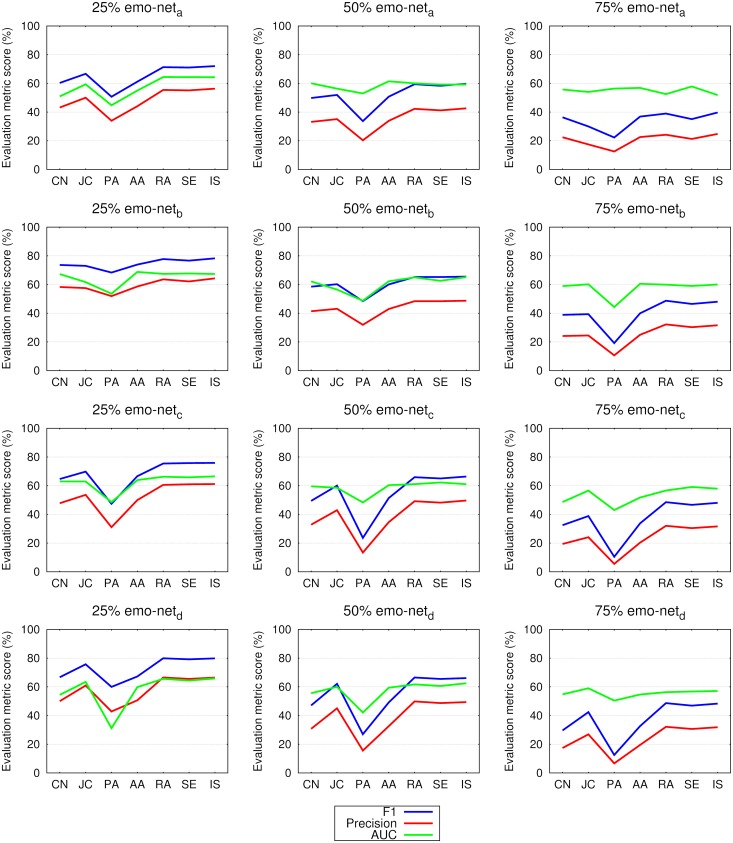
Link prediction in 25%, 50% and 75% of the links in networks constructed from all the words in tweets of the emo-net^*a*^, emo-net^*b*^, emo-net^*c*^ and emo-net^*d*^ datasets. Shown are the evaluation metric scores (see legend), namely the F1 score, the precision, and the area under the receiver operating characteristic curve (AUC), as obtained for seven different link prediction measures, namely common neighbors (CN), the Jaccard coefficient (JC), preferential attachment (PA), Adamic-Adar (AA), the resource allocation index (RA), selectivity (SE) and inverse selectivity (IS). The values of the F1 score and of precision are decreasing with the longitudinal growth of the networks (from 25% to 75%), while the AUC does better at retaining values regardless of the used percentage of links. The PA link prediction measure exposes the lowest link prediction potential on the emo-net dataset, this is regardless of the evaluation metrics used. See Table 2 and the main text for details.

**Fig 3:**
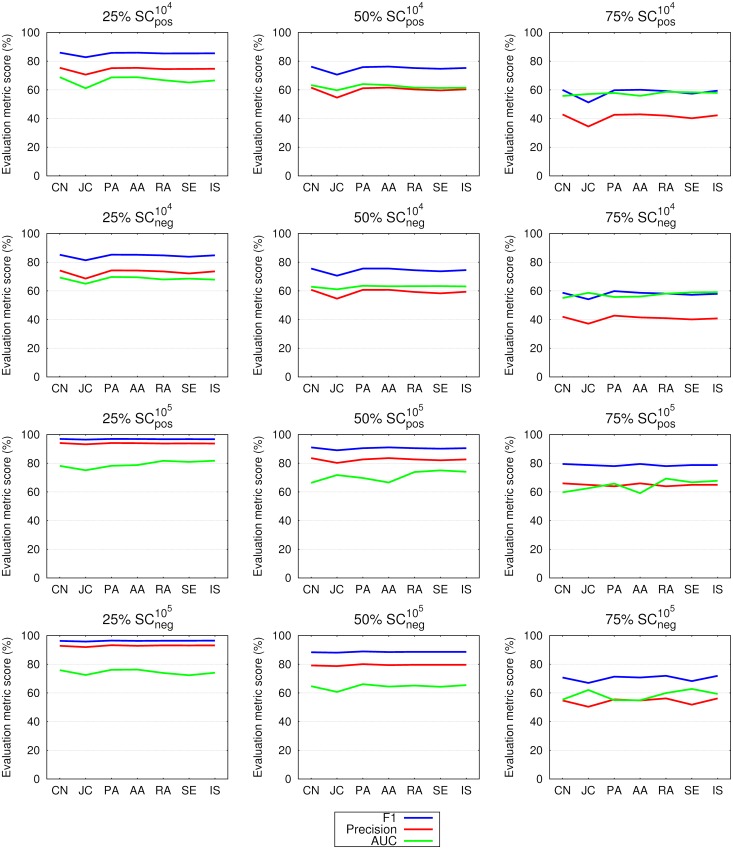
Link prediction in 25%, 50% and 75% of the links in networks constructed from all the words in tweets of the ${\text{SC}}_{neg}^{10^{4}}$, ${\text{SC}}_{pos}^{10^{4}}$, ${\text{SC}}_{neg}^{10^{5}}$ and ${\text{SC}}_{pos}^{10^{5}}$ datasets. Shown are the same quantities as in Fig 2. Here too the values of the F1 score and of precision are decreasing with the longitudinal growth of the networks (from 25% to 75%), while the AUC does better at retaining values regardless of the percentage of links used. It can also be observed that larger networks yield better link prediction measures. See Table 2 and the main text for details.

The link prediction results in networks constructed from all the words in tweets are presented in Fig 2 for the emo-net dataset, while Fig 3 shows the results for the SC dataset. In both figures the results are contrasted between precision, the F1 score and the area under the receiver operating characteristic curve (AUC). It can be observed that the F1 score and precision follow the same regularities i.e. exhibit decreasing values from the 25% to 75% networks regardless of the dataset. In emo-nets the weighted preferential attachment (PA) is systematically under-performing while the weighted Jaccard coefficient (JC) slightly deteriorates in the $\text{S}{\text{C}}^{10^{4}}$ datasets. The achieved results are in a favor of larger datasets. Also the difference between the F1 score and precision is lower in the SC datasets, especially in $\text{S}{\text{C}}^{10^{5}}$ and link prediction performance increases with the size of the data used. AUC exposes no substantial variability over different datasets, improvement is only noticed in larger in datasets ($\text{S}{\text{C}}^{10^{5}}$) regardless of the link prediction measure. From the presented results it is difficult to judge about the performance of the tested link prediction measures, therefore the analysis of ranking of seven link prediction measures follows.

**Fig 4:**
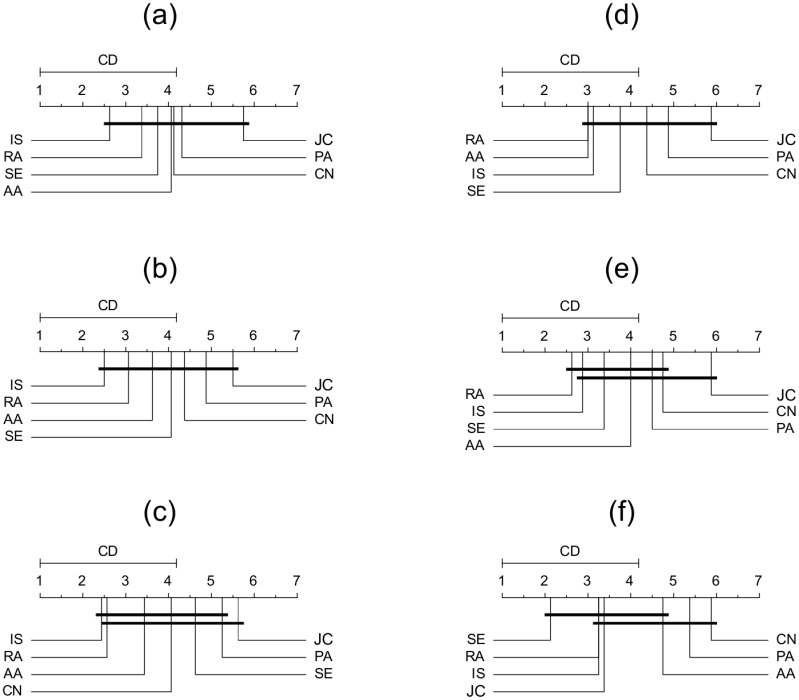
Ranking diagrams based on networks constructed from all the words in tweets for the seven link prediction measures used in this paper. Namely for common neighbors (CN), the Jaccard coefficient (JC), preferential attachment (PA), Adamic-Adar (AA), the resource allocation index (RA), selectivity (SE) and inverse selectivity (IS). Rankings according to the F1 score are presented on the left for 25% (a), 50% (b) and 75% (c), while rankings according to the area under the receiver operating characteristic curve (AUC) are presented on the right for 25% (d), 50% (e) and 75% (f). The best rank is at the leftmost position and the line below denotes measures which are not significantly different (Nemenyi test with *p*-values of 0.05).

In Fig 4 we show rank diagrams for the F1 score (left) and the area under the receiver operating characteristic curve (AUC) (right) for the 25% (top), 50% (middle) and 75% (bottom of the figure) networks from all-words in tweets over all datasets.

Rankings between precision (see data in S1 Text) and the F1 score are preserved for the 25% and 75% networks, while the rankings with AUC exhibit a different trend. Inverse selectivity (IS) is at the highest rank according to the F1 score, while AUC ranks the resource allocation index at the top position. Additionally, we consider the average overall rank across all networks (25%, 50% and 75%) of link prediction measures which positions at the top three places IS, AA, RA (according to the F1 score evaluation) and RA, SE and IS (according to the AUC evaluation).

### Link prediction results in hashtag networks

Next we analyze the difference between the hashtags’ networks compared to the all-words networks. Regardless of the tested measures or corpora, the results are only changed slightly–mainly deteriorated but in some cases also slightly improved.

**Fig 5:**
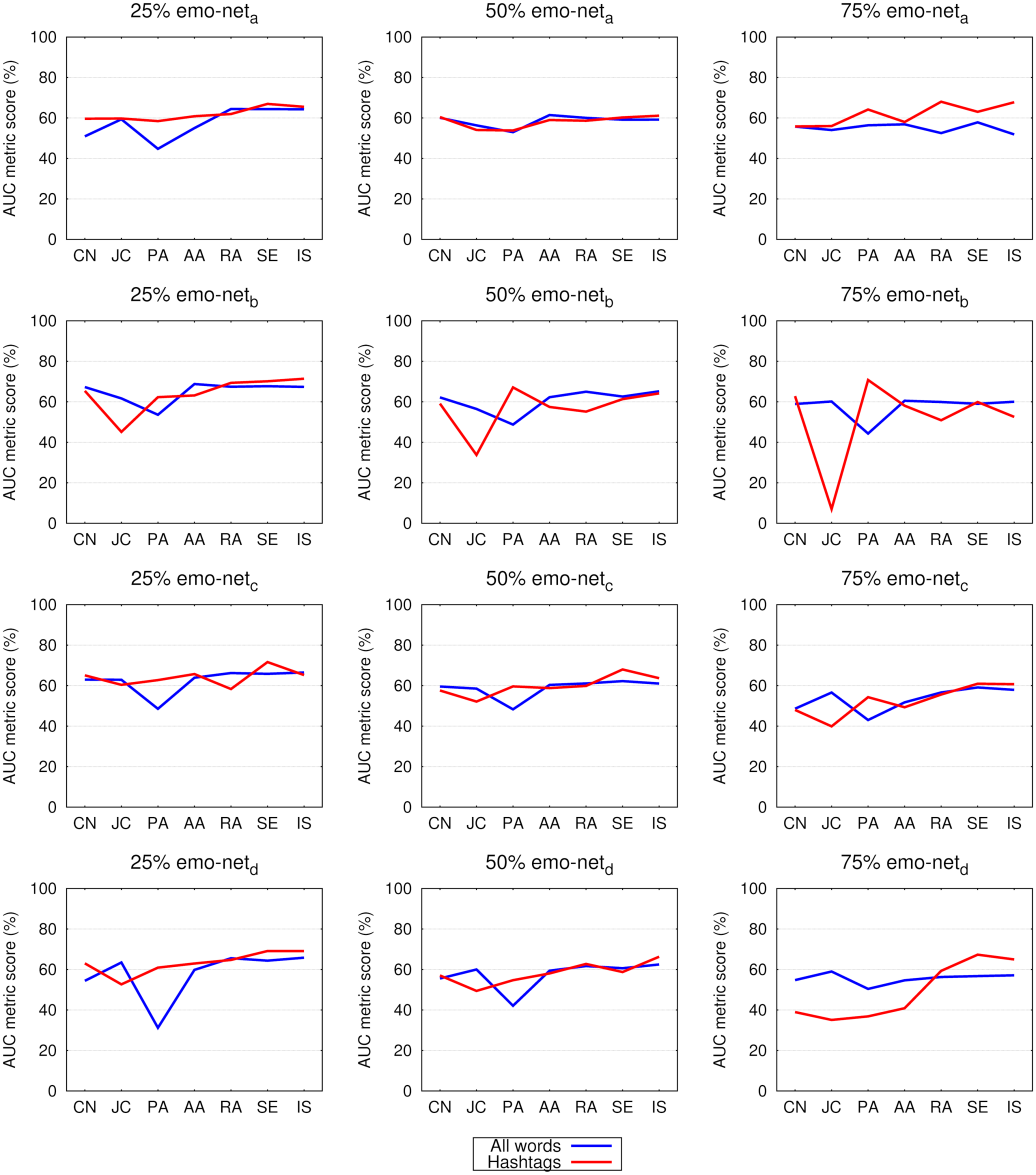
Link prediction in 25%, 50% and 75% of links in networks constructed from all the words and from hashtags (see legend) in tweets of the emo-net^*a*^, emo-net^*b*^, emo-net^*c*^ and emo-net^*d*^ datasets. Shown is the area under the receiver operating characteristic curve (AUC), as obtained for seven different link prediction measures, namely common neighbors (CN), the Jaccard coefficient (JC), preferential attachment (PA), Adamic-Adar (AA), the resource allocation index (RA), selectivity (SE) and inverse selectivity (IS). See Table 2 and the main text for details.

**Fig 6:**
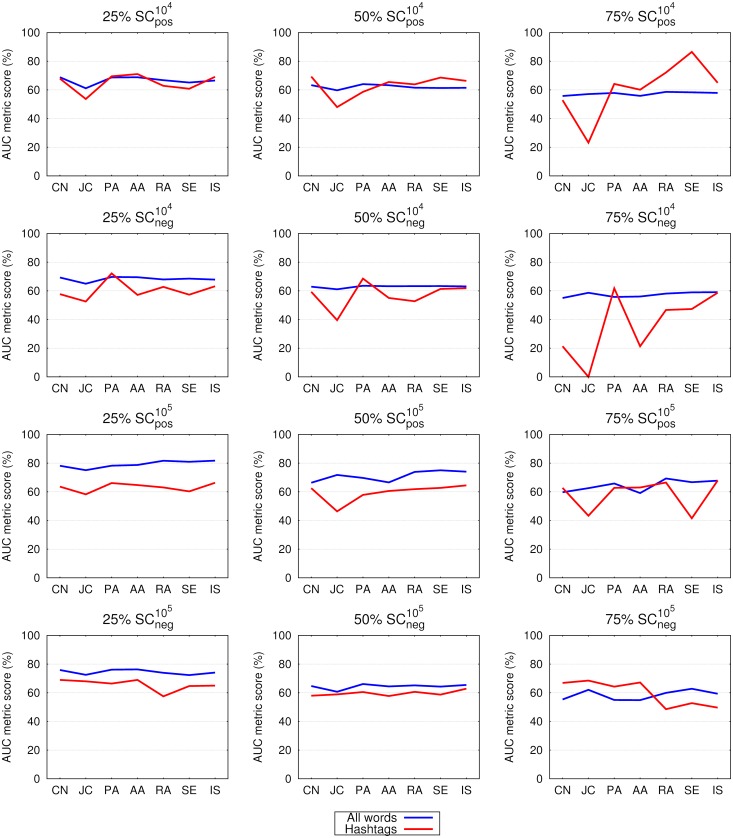
Link prediction in 25%, 50% and 75% of the links in networks constructed from all the words and from hashtags (see legend) in tweets of the ${\text{SC}}_{neg}^{10^{4}}$, ${\text{SC}}_{pos}^{10^{4}}$, ${\text{SC}}_{neg}^{10^{5}}$ and ${\text{SC}}_{pos}^{10^{5}}$ datasets. Shown are the same quantities as in Fig 5. See Table 2 and the main text for details.

Figs 5 and 6 compare the area under the receiver operating characteristic curve (AUC) values of the all-words and hashtags networks. If we consider the F1 score as an evaluation metric on smaller emo-net datasets, the results of all-words over the respective hashtag networks are improved by 13-37% (for the 25% networks); 11-30% (50% networks) and 8-21% (75% networks). On the SC dataset the results of the all-words’ networks are better by: 38-50% (25%); 43-53% (50%) and 35-54% (75%). In terms of AUC the observed differences are in general smaller: for emo-net up to 30% (25% networks); 19% (50%) and 22% (75%) and for the SC datasets up to 20% (25%); 15% (50%) and 25% (75%).

**Fig 7:**
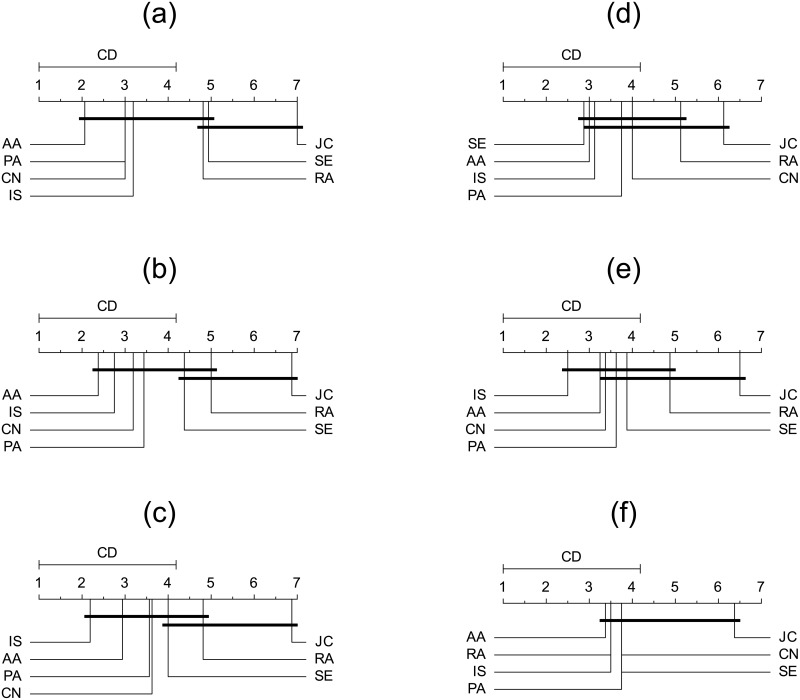
Ranking diagrams based on networks constructed from the hashtags in tweets for the seven link prediction measures used in this paper. Namely for common neighbors (CN), the Jaccard coefficient (JC), preferential attachment (PA), Adamic-Adar (AA), the resource allocation index (RA), selectivity (SE) and inverse selectivity (IS). Rankings according to the F1 score are presented on the left for 25% (a), 50% (b) and 75% (c), while rankings according to the area under the receiver operating characteristic curve (AUC) are presented on the right for 25% (d), 50% (e) and 75% (f). The best rank is at the leftmost position and the line below denotes measures which are not significantly different (Nemenyi test with *p*-values of 0.05).

Finally, the ranks are presented in Fig 7 for the hashtags’ networks of the 25%, 50% and 75% of the links for the F1 score (left) and AUC (right) respectively. The rank analysis reveals that the F1 score and AUC are interchanging Adamic-Adar, selectivity and inverse selectivity at the highest positions. The top overall average ranks achieved for the F1 score and AUC on the hashtags are: IS, AA, PA and IS, SE, PA respectively.

Alternative rankings according to different evaluation scores indicate the need for considering different evaluation metrics simultaneously, while using only one metric provides myopic insights into the results. This is strong evidence that multiple evaluation metrics should be considered for the evaluation of link prediction of the future content of tweets. The reported results also suggest that F1 score is a better candidate than precision, so for future research in link prediction in language networks we suggest considering the F1 score and AUC in parallel.

**Fig 8:**
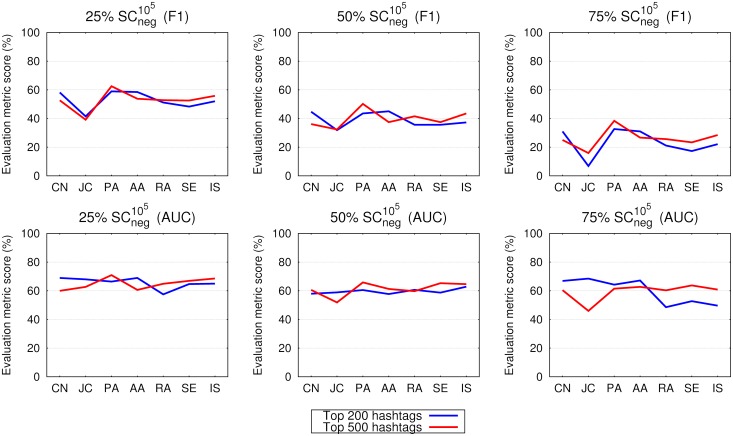
Link prediction in 25%, 50% and 75% of the links in networks constructed from the top 200 and top 500 hashtags (see legend) in tweets of the ${\text{SC}}_{neg}^{10^{5}}$ dataset. The upper row shows the F1 score, while the bottom row shows the area under the receiver operating characteristic curve (AUC), as obtained for the seven different link prediction measures considered in this paper.

Finally, we test whether the network construction principles of cutting off the top 200 most frequent words (hashtags) influences the obtained results. The construction of the top 500 hashtags’ networks follows the same principles except that the cut-off threshold is set to 500 instead of 200. The ${\text{SC}}_{pos}^{10^{5}}$ dataset was selected due to the sufficient number of different hashtags and the size of 10^5^. The results in Fig 8 depict the differences between the obtained top 200 and top 500 results in terms of the F1 and AUC scores for the 25%, 50% and 75% hashtags’ networks respectively. There are insignificant differences in the obtained results between the top 200 and the top 500 networks, except for the AUC from the 75% networks. AUC notably deteriorates in $\text{S}{\text{C}}^{10^{4}}$ 500 networks, due to the number of different hashtags below 160.

## Discussion

The trend of decreasing precisions and F1 score values along the 25% to 75% links in networks is present for all-words’ and hashtags’ networks. In networks created from 25% of the data, many probable links are left out. At the same time the most probable links are the most likely to be predicted and the link prediction measures are the most successful in predicting highly-probable links. With more data in the 50% and 75% networks the majority of highly-probable links are already included in the network, therefore the prediction measure is expected to predict less-probable links, which causes the drop in the prediction precision and the F1 score. At the same time AUC is prone to this effect. Zhao et al. in [45] observe similar problems in the dataset for testing, which they overcome by computing the odds ratio for correcting the prediction results. Following the same principle we plan to introduce the odds ratio into the evaluation of link prediction in language networks.

Regarding the size of the used datasets (10^5^ vs 10^4^ in SC) we can conclude that more data raise the improvement in the obtained results (as expected)—F1 scores are improved but the values of the area under the receiver operating characteristic curve (AUC) are of the same range and not notably higher. Hence, we can consider the results for the 10^4^ size as representative, especially when we regard the network construction principles being the same and resulting from networks of approximately the same size of nodes.

The F1 score and precision values shown in Figs 2 and 3 exhibit regularities across tested link prediction measures and datasets. The F1 score, calculated as the harmonic mean of precision and recall, is a more suitable evaluation metric than precision. Hence, we confirm the findings for social follower networks in [38], and for reciprocal follower networks on Twitter in [41] also for language networks constructed from the content of tweets—words and hashtags.

The two newly proposed measures for link prediction selectivity (SE) and inverse selectivity (IS) proved correct, especially IS which is ranked the best in 8 out of 18 cases, AA is the best 5 times, while SE and RA are at the top ranked position twice. In contrary JC occurred 17 times at the lowest rank. This is in accordance with other reported results where the measures which punish the nodes with a higher degree (AA, RA, SE and IS) are overperforming common neighbors, the Jaccard coefficient and preferential attachment in biological, social or technical networks [36, 43, 45]. Due to the achieved scores and low computational cost, we can conclude that selectivity and inverse selectivity should be considered for weighted link prediction, especially when dealing with texts in language networks.

Due to the same construction principles we analyse networks of a similar size, which is reflected on the very comparable results in hashtags to all-words’ networks. The network density is high and as expected systematically increasing from the 25% to 100% all-words’ networks, while hashtags’ networks exhibit some variations, especially in the SC dataset. Murata and Moriyasi in [43] discuss the positive influence of the network density on the performance of the weighted similarity measures, which is also reflected in our results. Next, all the studied networks are characterized by a relatively high average clustering coefficient, a very high average degree and average strength underpinning the efficiency of weighted similarity measures in both words’ and hashtags’ networks.

The area under the receiver operating characteristic curve (AUC) value of 0.5 is a random prediction—there is no relationship between the predicted values and the truth. An AUC below 0.5 indicates there is a relationship between the predicted values and the truth, but the model is backwards, i.e., predicts smaller values for positive cases. Another way to think of AUC is to imagine sorting the data by predicted values. Suppose this sort is not perfect, i.e., some positive cases sort below some negative cases, then AUC effectively measures how many times you would have to swap cases with their neighbors to repair the sort. Thus, sometimes we obtain a value below 0.5 for the weighted preferential attachment measure. All the networks have an assortativity between -0.02 and -0.52 which characterize the networks from the content of tweets as non-assortative. This is related to preferential attachment indicating that this is not the underlying mechanism for the growth of language networks. Finally, this is reflected in the score of preferential attachment with some AUC values below 0.5.

Link prediction is known to be an unbalanced classification problem and the receiver operating characteristic curves are insensitive to changes in class distributions and therefore insensitive to skewed class distributions [59]. Hence, it is no surprise that AUC metric provides more consistent insights into a measured performance over different datasets. Still, it would be wrong to neglect the F1 score for the evaluation since it provides a different perspective of the results. This is especially important, since we are dealing with text and hashtags. The content of microblogs represented in the form of words and hashtags is important for information representation and information propagation which are of interest in the information retrieval discipline as well. Information retrieval is traditionally oriented towards the F1 score based evaluations. Hence based on our findings we advocate the use of the F1 score and AUC simultaneously. To conclude, we find the introduced rank diagrams as a very useful tool which helps in merging the results of two or more evaluation metrics, and undoubtedly helps in gaining a holistic overview of the link prediction measures’ performance over different datasets.

In general hashtag networks exhibit similar characteristics as all-word networks: there is less difference of the AUC values than in terms of the F1 scores; hashtags constantly have lower F1 scores than all-words’ counterparts, while AUCs are of the same range. F1 scores are decreasing from the 25% to 75% networks, while AUC expose constant values; and there are no significant deviations in results on larger datasets. The only salient behaviour is noticed between the number of hashtags in the emo-net and SC datasets: it seems that the more recent tweeting trends rise more systematic (frequent) use of hashtags, which is reflected onto the structural properties of the studied networks. The influence of the distribution of hashtags per tweet is elaborated in [26] where they report about 50% of tweets tagged with one hashtag (dataset collected in 2013), while authors in [29] report around 15% of tweets with one hashtag (dataset collected before 2011). Next, the expansion of the network structure to the top 500 hashtags (Fig 8) exhibited no significant improvements. The importance of hashtags is reflected in capturing the semantic context of tweets, and as such are important for the summarization and categorization of the tweets’s content. This study is an initially step toward revealing the deeper structural properties of hashtags and will be addressed in our future studies.

## Conclusions

In this work we analysed link prediction based on the local similarity measures on networks constructed from the content of tweets: all-words and hashtags. The main goal of this analysis is to find which measure performs better in the task of predicting the future linking of words and hashtags in the content of tweets, which can be utilized for the propagation of information and opinion in social networks.

Besides five already analysed measures for link prediction in weighted complex networks of common neighbors (CN), the Jaccard coefficient (JC), preferential attachment (PA), Adamic-Adar (AA) and the resource allocation index (RA), we proposed two new measures: selectivity (SE) and inverse selectivity (IS). The experimental results obtained from two corpora of English tweets through the construction of systematically growing subnetworks form the 25%, 50% and 75% of the links and evaluated on the full content of 100% of the links in the network revealed many new findings.

First, the introduced ranking diagrams proved beneficial, as a powerful and straightforward tool for comparing the achieved scores of multiple tested link prediction measures on multiple datasets. The alternative rankings achieved by different evaluation scores (the F1 score and the area under the receiver operating characteristic curve) indicate the need to consider multiple evaluation metrics simultaneously, in order to obtain an unimpeded perspective on the link prediction on Twitter. Second, the two newly proposed measures selectivity (SE) and inverse selectivity (IS) proved efficient, especially IS, which is ranked best in 8 out of 18 cases, AA is the best 5 times, while SE and RA are at the top ranked position twice. In contrast, JC occurred 17 times at the lowest rank. Inverse selectivity is the first choice of measures for the task of predicting the future content of tweets. Third, the hashtags results exhibit similar characteristics as all-words networks, and as such are suitable candidates for the further examination of the content on Twitter within a complex network framework. Besides that, hashtags are able to capture the semantic context of tweets, and as such are important for the summarization and categorization of tweets.

The presented research reveals many possible direction for future studies. The focus of our future research plans is a deeper investigation of hashtag networks, incorporating the prediction of weights on the links and introducing the odds ratio to evaluate weighted link prediction in language networks.

## Supporting information

S1 Text Supplementary text for link prediction on Twitter. We provide additional details for all the measures used for the quantification of the studied networks, together with the definition of a standard set of network measures used for exploring their structure. Rankings for precision are provided as well.
(PDF)
