# Supplementary material for: Link prediction on Twitter
Source: PLoS One. 2017 Jul 18;12(7):e0181079. doi: 10.1371/journal.pone.0181079 (PMC5515441; doi:10.1371/journal.pone.0181079)
Supplement: S1 Text — We provide additional details for all the measures used for the quantification of the studied networks, together with the definition of a standard set of network measures used for exploring their structure. Rankings for precision are provided as well. (PDF) [file pone.0181079.s001.pdf]

Supplementary Material:
Link Prediction on Twitter

Sanda Martinčić-Ipšić, Edvin Močibob
smarti@uniri.hr, emocibob@student.uniri.hr
Department of Informatics, University of Rijeka, Rijeka, Croatia

Matjaž Perc
matjaz.perc@uni-mb.si
Faculty of Natural Sciences and Mathematics, University of Maribor, Maribor, Slovenia
Center for Applied Mathematics and Theoretical Physics, University of Maribor, Maribor, Slovenia

1 Introduction

This is the supplementary material for the paper "Link Prediction on Twitter". In the first Section we present the used network analysis measures and results obtained for the networks constructed from the content of tweets. In the second Section we list the results for seven link prediction measures.

2 Network Analysis Measures and Results

Initially we present the characterization of networks with the standard set of network measures. Table 1 in the supplementary material shows the results for *emo-net* datasets while Table 2 shows the results for *SC* datasets. The characterization of hashtags networks is in Tab. 3 for *emo-net* and in Tab. 4 for *SC* datasets. All results are reported for the full 100% of links in networks and for subnetworks with the 75%, 50% and 25% of links.

For the characterization of complex networks we use measures defined in the continuation. The network $G = (V, E)$ is a pair of a set of nodes V (or vertices) and a set of links E (or edges), where N is the number of nodes and K is the number of links. The number of network components is denoted by ω. In weighted networks every link connecting two nodes u and v has an associated weight w_{uv}. A node degree $deg(u)$ is the number of links directly connected (or incident) to node u and the set of nodes incident to a node u is denoted as $\Gamma(u)$. The strength of a node s_u is the sum of weights of all links incident to u.

Average network strength $\langle k \rangle$ is the sum of all link weights in a network divided by the number of nodes N:

$$\langle s \rangle = \frac{\sum_{u,v \in V} w_{uv}}{N}. \quad (1)$$

The average network degree $\langle k \rangle$ is the ratio of the number of links to the number of nodes. For undirected networks we multiply this ratio by 2 since undirected links always have two incident nodes:

$$\langle k \rangle = 2\frac{K}{N}. \quad (2)$$

Node selectivity, originally proposed by Masucci and Rodgers in 2006, for a node v corresponds to the sum of weights of all incident links divided by that nodes degree (denoted as $\deg(v)$):

$$e(v) = \frac{\sum_{u \in \Gamma(v)} w_{uv}}{\deg(v)}. \quad (3)$$

Network density is represented as the ratio between the number of existing links and the number of all possible links:

$$d = \frac{K}{N(N-1)}. \quad (4)$$

Average path length for a network, where d_{uv} denotes the number of links lying on the shortest path between $u, v \in V$, is computed as following:

$$L = \sum_{u,v} \frac{d_{uv}}{N(N-1)}. \quad (5)$$

The network diameter represents the longest shortest path in a network $(u, v \in V)$:

$$D = \max(d_{uv}). \tag{6}$$

The network radius denotes the shortest $\epsilon(v)$, where $\epsilon(v)$ is defined as the maximum distance between $v \in V$ and any other node:

$$R = \min(\epsilon(v)). \tag{7}$$

Network transitivity where possible triangles are identified by the number of triads (two links with a shared node):

$$T = 3\frac{\#triangles}{\#triads}. \tag{8}$$

Average clustering coefficient, where $c(v)$ is the clustering coefficient for a node v, sums all the individual clustering coefficients and divides them by the number of nodes:

$$C = \frac{1}{N}\sum_{v \in V} c(v). \tag{9}$$

The global network efficiency is the reciprocal value of a networks average path length:

$$E = \frac{1}{L}. \tag{10}$$

The assortativity coefficient is the Pearson correlation coefficient of degree between pairs of linked nodes

$$A = \frac{\sum_{uv} uv(e_{uv} - a_u b_v)}{\sigma_a \sigma_b} \tag{11}$$

where e_{uv} is the joint probability distribution (mixing matrix) of the degrees where a_u and b_v are the fraction of links that start and end at nodes u and v, and where σ_a and σ_b are the standard deviations of the distributions a_u and b_v. Here we present the sum rules which e_{uv} satisfies:

$$\sum_{uv} e_{uv} = 1, \quad \sum_{v} e_{uv} = a_u, \quad \sum_{u} e_{uv} = b_v. \tag{12}$$

Table 1 presents the network measures for *emo-net* datasets constructed as co-occurrence of **all-words** in tweets. The results are listed for subnetworks constructed from the 25%, 50%, 75% and 100% of links.

Table 2 presents the network measures for *SC* datasets constructed as the co-occurrence of **all-words** in tweets. The results are listed for subnetworks constructed from the 25%, 50%, 75% and 100% of links.

Table 3 presents the network measures for *emo-net* datasets constructed as the co-occurrence of **hashtags** in tweets. The results are listed for subnetworks constructed from the 25%, 50%, 75% and 100% of links.

Table 4 presents the network measures for *SC* datasets constructed as the co-occurrence of **hashtags** in tweets. The results are listed for subnetworks constructed from 25%, 50%, 75% and 100% of links.

2.1 Data

During the data preprocesing stage we removed stopwords acoording to the list available at www.ranks.nl/stopwords, extended with: ’rt’, ’-’, ’&’, ”, ’u’, ”, ’im’, ’!’, ’ur’, ’.’, ’4’, ’2’, ’@’, ’r’, ’?’, ’..’, ’...’.

The raw *emo-net* dataset separated into four parts and collected in February 2016 is available at http://langnet.uniri.hr/resources.html.

The *SC* dataset prepared in 2009 and annotated for polarity is available at http://help.sentiment140.com/for-students/.

Table 1: **The results for *emo-net* networks: co-occurrence of all-words in tweets.**

**100%**	$emo-net^a$	$emo-net^b$	$emo-net^c$	$emo-net^d$
N	201	200	201	204
K	10091	11289	9537	12787
$\langle k \rangle$	100.408	112.89	94.8955	125.3627
$\langle s \rangle$	995.8706	790.42	1069.4378	963.549
$\langle e \rangle$	21.8876	16.6454	29.1956	21.3748
d	0.502	0.5673	0.4745	0.6176
ω	1	1	1	1
L	1.498	1.4349	1.5255	1.3827
D	2	3	2	3
R	1	2	1	2
T	0.6916	0.7581	0.7124	0.7701
C	0.0073	0.0132	0.0047	0.0088
A	-0.1713	-0.1539	-0.1726	-0.1183
E	0.6676	0.6969	0.6555	0.7232
**75%**	$emo-net^b$	$emo-net^b$	$emo-net^c$	$emo-net^d$
N	201	200	199	204
K	9405	10460	8794	11798
$\langle k \rangle$	93.5821	104.6	88.3819	115.6667
d	0.4679	0.5256	0.4464	0.5698
T	0.6667	0.7225	0.6786	0.7347
C	0.0086	0.0141	0.0048	0.0099
**50%**	$emo-net^b$	$emo-net^b$	$emo-net^c$	$emo-net^d$
N	201	193	198	204
K	8270	9196	7715	10395
$\langle k \rangle$	82.2886	95.2953	77.9293	101.9118
d	0.4114	0.4963	0.3956	0.502
T	0.6243	0.6796	0.6287	0.6818
C	0.0106	0.0148	0.0052	0.0111
**25%**	$emo-net^b$	$emo-net^b$	$emo-net^c$	$emo-net^d$
N	201	191	196	204
K	6581	7070	6102	7947
$\langle k \rangle$	65.4826	74.0314	62.2653	77.9118
d	0.3274	0.3896	0.3193	0.3838
T	0.5526	0.5927	0.5538	0.5818
C	0.0165	0.0181	0.0059	0.0135

The results are expressed for N: number of nodes, K: number of links, $\langle k \rangle$: average network degree, $\langle s \rangle$: average network strength, $\langle e \rangle$: average network selectivity, d: network density, ω: number of components, L: average path length, D: network diameter, R: network radius, T: network transitivity, C: average clustering coefficient, A: network degree assortativity (not weighted) and E: global network efficiency. Measurements are reported for the 25%, 50%, 75% and 100% subnetworks of the four *emo-net* datasets of the same size - 10000 tweets.

Table 2: **The results for SC networks: co-occurrence of all-words in tweets.**

**100%**	$SC^{10^4}_{neg}$	$SC^{10^4}_{pos}$	$SC^{10^5}_{neg}$	$SC^{10^5}_{pos}$
N	200	200	200	200
K	16335	15666	19813	19711
$\langle k \rangle$	163.35	156.66	198.13	197.11
$\langle s \rangle$	720.06	694.14	8727.625	8329.75
$\langle e \rangle$	7.9847	7.83	87.8059	83.8993
d	0.8209	0.7872	0.9956	0.9905
ω	1	1	1	1
L	1.1791	1.2128	1.0044	1.0095
D	2	2	2	2
R	1	1	1	1
T	0.8564	0.8413	0.9958	0.9917
C	0.0066	0.0056	0.0024	0.0023
A	-0.1247	-0.1446	-0.0245	-0.0282
E	0.8481	0.8246	0.9956	0.9906
**75%**	$SC^{10^4}_{neg}$	$SC^{10^4}_{pos}$	$SC^{10^5}_{neg}$	$SC^{10^5}_{pos}$
N	200	200	200	200
K	15131	14519	19716	19574
$\langle k \rangle$	151.31	145.19	197.16	195.74
d	0.7604	0.7296	0.9908	0.9836
T	0.8119	0.7997	0.9913	0.986
C	0.007	0.006	0.0024	0.0022
**50%**	$SC^{10^4}_{neg}$	$SC^{10^4}_{pos}$	$SC^{10^5}_{neg}$	$SC^{10^5}_{pos}$
N	200	200	200	200
K	13367	12706	19484	19274
$\langle k \rangle$	133.67	127.06	194.84	192.74
d	0.6717	0.6385	0.9791	0.9685
T	0.7505	0.74	0.9809	0.9736
C	0.008	0.0068	0.0024	0.0023
**25%**	$SC^{10^4}_{neg}$	$SC^{10^4}_{pos}$	$SC^{10^5}_{neg}$	$SC^{10^5}_{pos}$
N	200	200	200	200
K	10195	9810	18693	18129
$\langle k \rangle$	101.95	98.1	186.93	181.29
d	0.5123	0.493	0.9393	0.911
T	0.6416	0.6402	0.9476	0.9304
C	0.0101	0.0082	0.0024	0.0023

The results are expressed for N: number of nodes, K: number of links, $\langle k \rangle$: average network degree, $\langle s \rangle$: average network strength, $\langle e \rangle$: average network selectivity, d: network density, ω: number of components, L: average path length, D: network diameter, R: network radius, T: network transitivity, C: average clustering coefficient, A: network degree assortativity (not weighted) and E: global network efficiency. Measurements are reported for 25%, 50%, 75% and 100% subnetworks of the four SC datasets. The SC^{10^4} networks are constructed from 10000 tweets, while SC^{10^5} are constructed from 100000 tweets.

Table 3: **The results for *emo-net* networks: co-occurrence of hashtags in tweets.**

**100%**	$emo-net^a$	$emo-net^b$	$emo-net^c$	$emo-net^d$
N	199	200	197	202
K	3113	1005	1971	1717
$\langle k \rangle$	31.2864	10.05	20.0102	17.0
$\langle s \rangle$	276.7889	48.29	111.6142	97.9752
$\langle e \rangle$	10.9415	4.2814	5.4212	5.5559
d	0.158	0.0505	0.1021	0.0846
ω	1	1	1	1
L	1.8707	2.2662	2.0681	1.9989
D	3	4	3	4
R	2	2	2	2
T	0.4184	0.2498	0.4126	0.3215
C	0.0054	0.0061	0.0089	0.0059
A	-0.3454	-0.4222	-0.3778	-0.4408
E	0.5346	0.4413	0.4835	0.5003
**75%**	$emo-net^b$	$emo-net^b$	$emo-net^c$	$emo-net^d$
N	199	193	194	194
K	2887	903	1787	1521
$\langle k \rangle$	29.0151	9.3575	18.4227	15.6804
d	0.1465	0.0487	0.0955	0.0812
T	0.4004	0.2478	0.3954	0.3132
C	0.0057	0.007	0.0092	0.0065
**50%**	$emo-net^b$	$emo-net^b$	$emo-net^c$	$emo-net^d$
N	188	173	182	185
K	2462	757	1459	1271
$\langle k \rangle$	26.1915	8.7514	16.033	13.7405
d	0.1401	0.0509	0.0886	0.0747
T	0.3911	0.2502	0.3766	0.3041
C	0.0068	0.0085	0.0115	0.0079
**25%**	$emo-net^b$	$emo-net^b$	$emo-net^c$	$emo-net^d$
N	178	132	147	159
K	1825	497	1054	904
$\langle k \rangle$	20.5056	7.5303	14.3401	11.3711
d	0.1159	0.0575	0.0982	0.072
T	0.3512	0.2583	0.3736	0.2904
C	0.0109	0.0122	0.0221	0.0127

The results are expressed for N: number of nodes, K: number of links, $\langle k \rangle$: average network degree, $\langle s \rangle$: average network strength, $\langle e \rangle$: average network selectivity, d: network density, ω: number of components, L: average path length, D: network diameter, R: network radius, T: network transitivity, C: average clustering coefficient, A: network degree assortativity (not weighted) and E: global network efficiency. Measurements are reported for the 25%, 50%, 75% and 100% hastags subnetworks of the four *emo-net* datasets.

Table 4: **The results for SC networks: co-occurrence of hashtags in tweets.**

**100%**	$SC^{10^4}_{neg}$	$SC^{10^4}_{pos}$	$SC^{10^5}_{neg}$	$SC^{10^5}_{pos}$
N	106	123	165	168
K	354	361	1169	1327
$\langle k \rangle$	6.6792	5.8699	14.1697	15.7976
$\langle s \rangle$	14.3868	14.5122	128.4545	157.2798
$\langle e \rangle$	1.88	1.8002	5.3122	5.6837
d	0.0636	0.0481	0.0864	0.0946
ω	3	4	3	1
L	2.4712	2.4764	2.1394	2.0799
D	5	5	4	4
R	3	3	2	2
T	0.304	0.2564	0.3788	0.3585
C	0.0137	0.0131	0.0025	0.0018
A	-0.3854	-0.4735	-0.4892	-0.5213
E	0.4047	0.4038	0.4674	0.4808
**75%**	$SC^{10^4}_{neg}$	$SC^{10^4}_{pos}$	$SC^{10^5}_{neg}$	$SC^{10^5}_{pos}$
N	85	99	144	156
K	274	299	1012	1146
$\langle k \rangle$	6.4471	6.0404	14.0556	14.6923
d	0.0768	0.0616	0.0983	0.0948
T	0.328	0.2812	0.398	0.3668
C	0.0201	0.0154	0.0029	0.002
**50%**	$SC^{10^4}_{neg}$	$SC^{10^4}_{pos}$	$SC^{10^5}_{neg}$	$SC^{10^5}_{pos}$
N	73	76	127	128
K	217	221	841	907
$\langle k \rangle$	5.9452	5.8158	13.2441	14.1719
d	0.0826	0.0775	0.1051	0.1116
T	0.3728	0.3041	0.3993	0.3791
C	0.0254	0.0239	0.0038	0.0027
**25%**	$SC^{10^4}_{neg}$	$SC^{10^4}_{pos}$	$SC^{10^5}_{neg}$	$SC^{10^5}_{pos}$
N	59	63	103	110
K	142	158	576	668
$\langle k \rangle$	4.8136	5.0159	11.1845	12.1455
d	0.083	0.0809	0.1097	0.1114
T	0.3314	0.3149	0.4151	0.3756
C	0.0312	0.0312	0.0052	0.0037

The results are expressed for N: number of nodes, K: number of links, $\langle k \rangle$: average network degree, $\langle s \rangle$: average network strength, $\langle e \rangle$: average network selectivity, d: network density, ω: number of components, L: average path length, D: network diameter, R: network radius, T: network transitivity, C: average clustering coefficient, A: network degree assortativity (not weighted) and E: global network efficiency). Measurements are reported for the 25%, 50%, 75% and 100% hastags subnetworks of the four SC datasets. The SC^{10^4} networks are constructed from 10000 tweets, while SC^{10^5} are constructed from 100000 tweets.

3 Results

3.1 Ranking diagrams for precision in all-words networks

In Fig. 1 of the supplementary materials we show ranking diagrams for precision for the the 25% (a), 50% (b) and 75% (c) networks from **all-words** in tweets over all datasets. The seven tested link prediction measures the weighted common neighbors (CN), the weighted Jaccard coefficient (JC), the weighted preferential attachment (PA), the weighted Adamic-Adar (AA), the resource allocation index (RA), selectivity (SE) and inverse selectivity (IS) are ranked according to the values of precision over eight datasets.

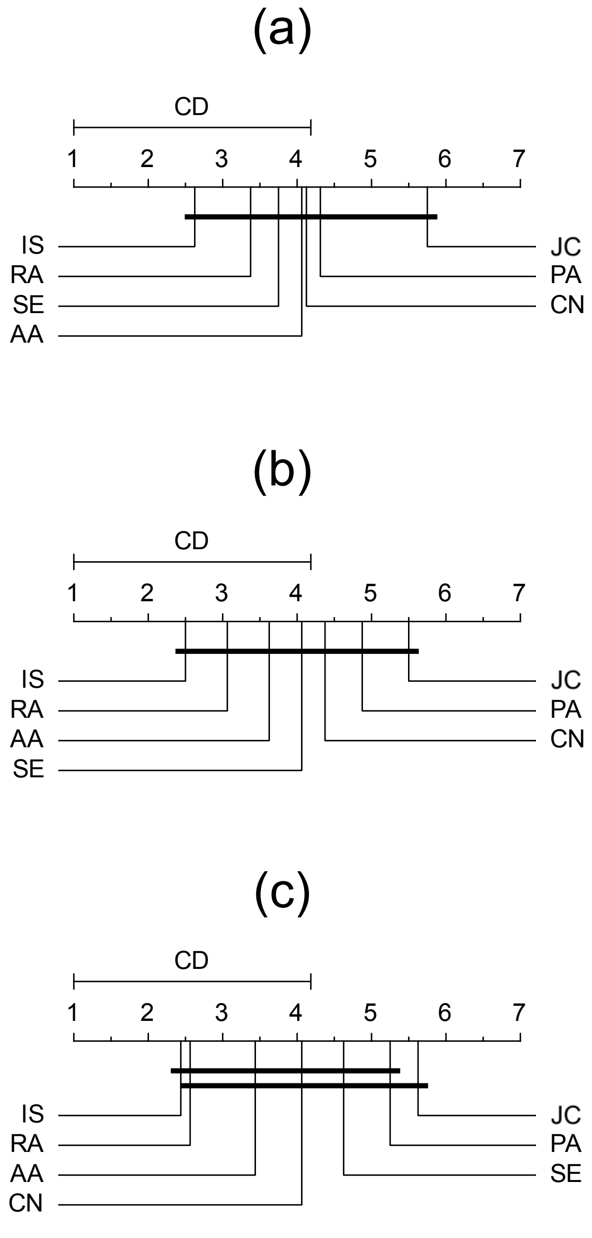

Figure 1: **Ranking diagrams** for seven link prediction measures: common neighbors (CN), the Jaccard coefficient (JC), preferential attachment (PA), Adamic-Adar (AA), the resource allocation index (RA), selectivity (SE) and inverse selectivity (IS). Rankings according to values of **precision** are presented for the 25% (a), 50% (b) and 75% (c) networks constructed for **all-words** in tweets. The best rank is at the far most left position and the line below denotes measures which are not significantly different (Nemenyi test with p-values below 0.05).

3.2 Ranking diagrams for precision in hastags networks

In Fig. 2 of the supplementary materials we show ranking diagrams for precision for the 25% (top), 50% (middle) and 75% (bottom) networks from **hastags** in tweets over all datasets. The seven tested link prediction measures the weighted common neighbors (CN), the weighted Jaccard coefficient (JC), the weighted preferential attachment (PA), the weighted Adamic-Adar (AA), the resource allocation index (RA), selectivity (SE) and inverse selectivity (IS) are ranked according to the values of precision over eight datasets.

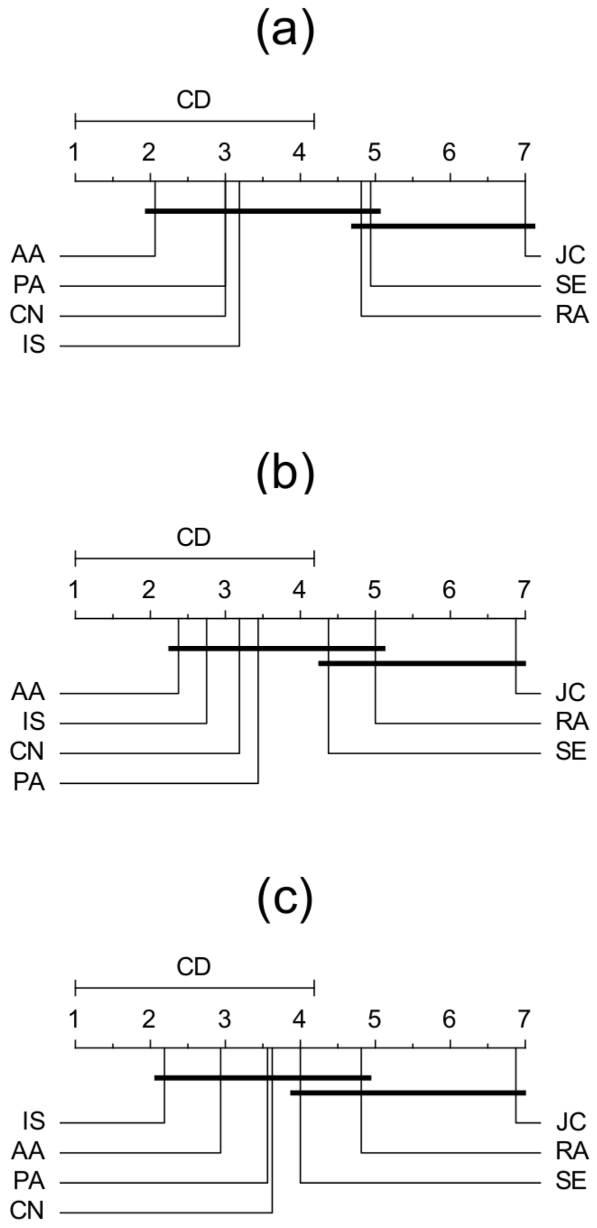

Figure 2: **Ranking diagrams** for seven link prediction measures: common neighbors (CN), the Jaccard coefficient (JC), preferential attachment (PA), Adamic-Adar (AA), the resource allocation index (RA), selectivity (SE) and inverse selectivity (IS). Rankings according to values of **precision** are presented for the 25% (a), 50% (b) and 75% (c) networks constructed for **hashtags** in tweets. The best rank is at the far most left position and the line below denotes measures which are not significantly different (Nemenyi test with p-values below 0.05).

3.3 Results for the networks from all-words in tweets

In this section we list results for the 25%, 50% and 75% networks constructed for co-occurance of all-words in tweets in all eight datasets. The results are reported for precision, the F1 score and the the area under the receiver operating characteristic curve (AUC) for each of the tested link prediction measures: the weighted common neighbors in Table 5, the weighted Jaccard coefficient in Table 6, the weighted preferential attachment in Table 7, the weighted Adamic-Adar in Table 8, the resource allocation index in Table 9, selectivity in Table 10 and inverse selectivity in Table 11.

Table 5: **PRECISION, F1 score and AUC for the weighted common neighbors**

**PRECISION**	$emo-net^a$	$emo-net^b$	$emo-net^c$	$emo-net^d$	$SC^{10^4}_{neg}$	$SC^{10^4}_{pos}$	$SC^{10^5}_{neg}$	$SC^{10^5}_{pos}$
25%	43.19%	58.31%	47.77%	50.02%	75.41%	74.23%	94.11%	92.86%
50%	33.17%	41.38%	32.82%	30.77%	61.59%	60.78%	83.59%	79.18%
75%	22.45%	24.13%	19.38%	17.39%	42.86%	41.59%	65.98%	54.74%
**F1**	$emo-net^a$	$emo-net^b$	$emo-net^c$	$emo-net^d$	$SC^{10^4}_{neg}$	$SC^{10^4}_{pos}$	$SC^{10^5}_{neg}$	$SC^{10^5}_{pos}$
25%	60.33%	73.66%	64.66%	66.69%	85.98%	85.21%	96.96%	96.3%
50%	49.81%	58.53%	49.42%	47.06%	76.23%	75.6%	91.06%	88.38%
75%	36.67%	38.87%	32.47%	29.63%	60.0%	58.74%	79.5%	70.75%
**AUC**	$emo-net^a$	$emo-net^b$	$emo-net^c$	$emo-net^d$	$SC^{10^4}_{neg}$	$SC^{10^4}_{pos}$	$SC^{10^5}_{neg}$	$SC^{10^5}_{pos}$
25%	50.93%	67.3%	62.99%	54.41%	68.85%	69.32%	78.17%	75.86%
50%	60.1%	62.19%	59.58%	55.56%	63.35%	62.92%	66.34%	64.71%
75%	55.74%	58.92%	48.62%	54.77%	55.76%	55.02%	59.71%	55.33%

The results in terms of precision, the F1 score and AUC for the weighted common neighbors for the 25%, 50% and 75% of links in all-words networks on eight datasets.

Table 6: **PRECISION, F1 score and AUC for the weighted Jaccard coefficient**

**PRECISION**	$emo-net^a$	$emo-net^b$	$emo-net^c$	$emo-net^d$	$SC^{10^4}_{neg}$	$SC^{10^4}_{pos}$	$SC^{10^5}_{neg}$	$SC^{10^5}_{pos}$
25%	50.03%	57.53%	53.62%	60.93%	70.64%	68.61%	93.21%	91.97%
50%	35.09%	43.1%	42.92%	45.03%	54.62%	54.59%	80.24%	78.72%
75%	17.64%	24.49%	24.09%	26.9%	34.47%	37.14%	64.95%	50.36%
**F1**	$emo-net^a$	$emo-net^b$	$emo-net^c$	$emo-net^d$	$SC^{10^4}_{neg}$	$SC^{10^4}_{pos}$	$SC^{10^5}_{neg}$	$SC^{10^5}_{pos}$
25%	66.69%	73.04%	69.81%	75.72%	82.79%	81.39%	96.49%	95.82%
50%	51.95%	60.23%	60.06%	62.09%	70.65%	70.63%	89.04%	88.09%
75%	29.99%	39.34%	38.83%	42.39%	51.27%	54.16%	78.75%	66.99%
**AUC**	$emo-net^a$	$emo-net^b$	$emo-net^c$	$emo-net^d$	$SC^{10^4}_{neg}$	$SC^{10^4}_{pos}$	$SC^{10^5}_{neg}$	$SC^{10^5}_{pos}$
25%	59.36%	61.68%	62.91%	63.47%	61.15%	65.0%	75.11%	72.52%
50%	56.35%	56.47%	58.55%	60.01%	59.72%	61.1%	71.78%	60.72%
75%	54.05%	60.16%	56.6%	59.01%	57.1%	58.66%	62.56%	62.06%

The results in terms of precision, the F1 score and AUC for the weighted Jaccard coefficient for the 25%, 50% and 75% of the links in all-words networks on eight datasets.

Table 7: **PRECISION, F1 score and AUC for the weighted preferential attachment**

**PRECISION**	$emo-net^a$	$emo-net^b$	$emo-net^c$	$emo-net^d$	$SC_{neg}^{10^4}$	$SC_{pos}^{10^4}$	$SC_{neg}^{10^5}$	$SC_{pos}^{10^5}$
25%	33.96%	51.93%	31.06%	42.83%	75.16%	74.3%	94.11%	93.3%
50%	20.32%	31.92%	13.39%	15.59%	61.15%	60.74%	82.67%	80.09%
75%	12.54%	10.62%	5.52%	6.67%	42.61%	42.72%	63.92%	55.47%
**F1**	$emo-net^a$	$emo-net^b$	$emo-net^c$	$emo-net^d$	$SC_{neg}^{10^4}$	$SC_{pos}^{10^4}$	$SC_{neg}^{10^5}$	$SC_{pos}^{10^5}$
25%	50.7%	68.36%	47.4%	59.97%	85.82%	85.26%	96.96%	96.53%
50%	33.77%	48.39%	23.62%	26.98%	75.89%	75.58%	90.52%	88.95%
75%	22.28%	19.19%	10.46%	12.51%	59.76%	59.87%	77.99%	71.36%
**AUC**	$emo-net^a$	$emo-net^b$	$emo-net^c$	$emo-net^d$	$SC_{neg}^{10^4}$	$SC_{pos}^{10^4}$	$SC_{neg}^{10^5}$	$SC_{pos}^{10^5}$
25%	44.75%	53.6%	48.56%	31.21%	68.71%	69.71%	78.23%	76.14%
50%	52.99%	48.76%	48.31%	42.11%	64.04%	63.58%	69.7%	66.11%
75%	56.4%	44.33%	43.02%	50.42%	57.87%	55.75%	65.81%	54.98%

The results in terms of precision, the F1 score and AUC for the weighted preferential attachment for the 25%, 50% and 75% of links in all-words networks on eight datasets.

Table 8: **PRECISION, F1 score and AUC for the weighted Adamic-Adar**

**PRECISION**	$emo-net^a$	$emo-net^b$	$emo-net^c$	$emo-net^d$	$SC_{neg}^{10^4}$	$SC_{pos}^{10^4}$	$SC_{neg}^{10^5}$	$SC_{pos}^{10^5}$
25%	44.1%	58.64%	49.99%	50.64%	75.37%	74.21%	94.02%	92.86%
50%	33.99%	42.95%	34.58%	32.57%	61.62%	60.78%	83.59%	79.41%
75%	22.59%	24.97%	20.32%	19.51%	42.94%	41.5%	65.98%	54.74%
**F1**	$emo-net^a$	$emo-net^b$	$emo-net^c$	$emo-net^d$	$SC_{neg}^{10^4}$	$SC_{pos}^{10^4}$	$SC_{neg}^{10^5}$	$SC_{pos}^{10^5}$
25%	61.21%	73.93%	66.65%	67.23%	85.96%	85.2%	96.92%	96.3%
50%	50.74%	60.09%	51.39%	49.13%	76.26%	75.6%	91.06%	88.52%
75%	36.86%	39.96%	33.78%	32.66%	60.08%	58.66%	79.5%	70.75%
**AUC**	$emo-net^a$	$emo-net^b$	$emo-net^c$	$emo-net^d$	$SC_{neg}^{10^4}$	$SC_{pos}^{10^4}$	$SC_{neg}^{10^5}$	$SC_{pos}^{10^5}$
25%	55.01%	68.79%	63.93%	59.82%	68.84%	69.52%	78.73%	76.29%
50%	61.46%	62.28%	60.38%	59.41%	63.28%	63.24%	66.57%	64.44%
75%	56.86%	60.52%	51.75%	54.66%	55.85%	56.06%	59.14%	54.88%

The results in terms of precision, the F1 score and AUC for the weighted Adamic-Adar for the 25%, 50% and 75% of links in all-words networks on eight datasets.

Table 9: **PRECISION, F1 score and AUC for the resource allocation index**

**PRECISION**	$emo-net^a$	$emo-net^b$	$emo-net^c$	$emo-net^d$	$SC^{10^4}_{neg}$	$SC^{10^4}_{pos}$	$SC^{10^5}_{neg}$	$SC^{10^5}_{pos}$
25%	55.44%	63.62%	60.58%	66.53%	74.53%	73.58%	93.75%	93.17%
50%	42.28%	48.4%	49.18%	49.83%	60.31%	59.26%	82.67%	79.63%
75%	24.2%	32.21%	32.03%	32.15%	42.03%	40.98%	63.92%	56.2%
**F1**	$emo-net^a$	$emo-net^b$	$emo-net^c$	$emo-net^d$	$SC^{10^4}_{neg}$	$SC^{10^4}_{pos}$	$SC^{10^5}_{neg}$	$SC^{10^5}_{pos}$
25%	71.33%	77.76%	75.45%	79.9%	85.41%	84.78%	96.77%	96.47%
50%	59.44%	65.23%	65.93%	66.52%	75.24%	74.42%	90.52%	88.66%
75%	38.97%	48.72%	48.52%	48.66%	59.18%	58.13%	77.99%	71.96%
**AUC**	$emo-net^a$	$emo-net^b$	$emo-net^c$	$emo-net^d$	$SC^{10^4}_{neg}$	$SC^{10^4}_{pos}$	$SC^{10^5}_{neg}$	$SC^{10^5}_{pos}$
25%	64.44%	67.45%	66.24%	65.57%	66.79%	67.96%	81.69%	73.93%
50%	60.04%	65.0%	61.09%	61.7%	61.55%	63.3%	73.89%	65.15%
75%	52.54%	59.91%	56.64%	56.3%	58.63%	58.11%	69.31%	59.94%

The results in terms of precision, the F1 score and AUC for the resource allocation index for the 25%, 50% and 75% of links in all-words networks on eight datasets.

Table 10: **PRECISION, F1 score and AUC for selectivity**

**PRECISION**	$emo-net^a$	$emo-net^b$	$emo-net^c$	$emo-net^d$	$SC^{10^4}_{neg}$	$SC^{10^4}_{pos}$	$SC^{10^5}_{neg}$	$SC^{10^5}_{pos}$
25%	55.13%	62.17%	60.99%	65.54%	74.59%	72.17%	93.84%	93.11%
50%	41.19%	48.4%	48.19%	48.7%	59.64%	58.31%	82.07%	79.63%
75%	21.28%	30.28%	30.42%	30.64%	40.2%	40.1%	64.95%	51.82%
**F1**	$emo-net^a$	$emo-net^b$	$emo-net^c$	$emo-net^d$	$SC^{10^4}_{neg}$	$SC^{10^4}_{pos}$	$SC^{10^5}_{neg}$	$SC^{10^5}_{pos}$
25%	71.07%	76.67%	75.77%	79.18%	85.45%	83.83%	96.82%	96.43%
50%	58.34%	65.23%	65.04%	65.5%	74.72%	73.67%	90.15%	88.66%
75%	35.1%	46.48%	46.65%	46.9%	57.35%	57.25%	78.75%	68.27%
**AUC**	$emo-net^a$	$emo-net^b$	$emo-net^c$	$emo-net^d$	$SC^{10^4}_{neg}$	$SC^{10^4}_{pos}$	$SC^{10^5}_{neg}$	$SC^{10^5}_{pos}$
25%	64.37%	67.72%	65.84%	64.38%	65.15%	68.55%	80.95%	72.34%
50%	59.16%	62.61%	62.24%	60.63%	61.34%	63.35%	75.02%	64.3%
75%	57.84%	59.02%	59.11%	56.75%	58.31%	58.96%	66.71%	62.8%

The results in terms of precision, the F1 score and AUC for selectivity for the 25%, 50% and 75% of links in all-words networks on eight datasets.

Table 11: **PRECISION, F1 score and AUC for inverse selectivity**

**PRECISION**	$emo-net^a$	$emo-net^b$	$emo-net^c$	$emo-net^d$	$SC^{10^4}_{neg}$	$SC^{10^4}_{pos}$	$SC^{10^5}_{neg}$	$SC^{10^5}_{pos}$
25%	56.32%	64.3%	61.16%	66.45%	74.71%	73.63%	93.75%	93.17%
50%	42.61%	48.73%	49.67%	49.37%	60.38%	59.39%	82.67%	79.63%
75%	24.78%	31.6%	31.63%	31.85%	42.28%	40.8%	64.95%	56.2%
**F1**	$emo-net^a$	$emo-net^b$	$emo-net^c$	$emo-net^d$	$SC^{10^4}_{neg}$	$SC^{10^4}_{pos}$	$SC^{10^5}_{neg}$	$SC^{10^5}_{pos}$
25%	72.06%	78.27%	75.9%	79.84%	85.52%	84.82%	96.77%	96.47%
50%	59.76%	65.53%	66.37%	66.11%	75.29%	74.52%	90.52%	88.66%
75%	39.72%	48.03%	48.06%	48.31%	59.43%	57.96%	78.75%	71.96%
**AUC**	$emo-net^a$	$emo-net^b$	$emo-net^c$	$emo-net^d$	$SC^{10^4}_{neg}$	$SC^{10^4}_{pos}$	$SC^{10^5}_{neg}$	$SC^{10^5}_{pos}$
25%	64.33%	67.37%	66.51%	65.83%	66.56%	67.87%	81.75%	74.1%
50%	59.19%	65.2%	61.04%	62.48%	61.47%	63.13%	74.05%	65.46%
75%	51.87%	60.04%	57.93%	57.13%	57.91%	59.08%	67.79%	59.33%

The results in terms of precision, the F1 score and AUC for inverse selectivity for the 25%, 50% and 75% of links in all-words networks on eight datasets.

3.4 Results for the networks from hashtags in tweets

In this section we list results for the 25%, 50% and 75% networks constructed for the co-occurance of **hashtags** in tweets in all eight datasets. The results are reported for precision, the F1 score and the AUC for each of the tested link prediction measures: the weighted common neighbors in Table 12, the weighted Jaccard coefficient in Table 13, the weighted preferential attachment in Table 14, the weighted Adamic-Adar in Table 15, the resource allocation index in Table 16, selectivity in Table 17 and inverse selectivity in Table 18.

Table 12: **PRECISION, F1 score and AUC for the weighted common neighbors**

**PRECISION**	$emo-net^a$	$emo-net^b$	$emo-net^c$	$emo-net^d$	$SC^{10^4}_{neg}$	$SC^{10^4}_{pos}$	$SC^{10^5}_{neg}$	$SC^{10^5}_{pos}$
25%	34.47%	26.57%	37.84%	35.79%	32.08%	31.53%	36.76%	42.64%
50%	23.35%	20.56%	28.52%	25.56%	17.52%	20.71%	23.78%	30.0%
75%	14.16%	11.76%	15.76%	13.27%	15.0%	9.68%	15.92%	20.44%
**F1**	$emo-net^a$	$emo-net^b$	$emo-net^c$	$emo-net^d$	$SC^{10^4}_{neg}$	$SC^{10^4}_{pos}$	$SC^{10^5}_{neg}$	$SC^{10^5}_{pos}$
25%	51.27%	41.99%	54.91%	52.72%	48.57%	47.94%	53.76%	59.79%
50%	37.86%	34.11%	44.38%	40.71%	29.81%	34.32%	38.42%	46.15%
75%	24.81%	21.05%	27.23%	23.42%	26.09%	17.65%	27.47%	33.94%
**AUC**	$emo-net^a$	$emo-net^b$	$emo-net^c$	$emo-net^d$	$SC^{10^4}_{neg}$	$SC^{10^4}_{pos}$	$SC^{10^5}_{neg}$	$SC^{10^5}_{pos}$
25%	59.64%	65.4%	65.59%	63.03%	68.7%	62.58%	64.77%	70.19%
50%	60.47%	59.11%	58.13%	57.06%	67.04%	65.56%	62.64%	61.24%
75%	55.84%	62.82%	48.01%	38.97%	60.72%	51.34%	63.12%	70.17%

The results in terms of precision, the F1 score and AUC for the weighted common neighbors for the 25%, 50% and 75%of links in hashtags networks on eight datasets.

Table 13: **PRECISION, F1 score and AUC for the weighted Jaccard coefficient**

**PRECISION**	$emo-net^a$	$emo-net^b$	$emo-net^c$	$emo-net^d$	$SC^{10^4}_{neg}$	$SC^{10^4}_{pos}$	$SC^{10^5}_{neg}$	$SC^{10^5}_{pos}$
25%	26.24%	10.43%	26.83%	15.13%	16.04%	12.81%	24.62%	25.19%
50%	19.2%	6.85%	20.9%	7.85%	6.57%	5.71%	17.99%	18.33%
75%	10.18%	1.96%	14.13%	2.04%	6.25%	3.23%	8.28%	3.31%
**F1**	$emo-net^a$	$emo-net^b$	$emo-net^c$	$emo-net^d$	$SC^{10^4}_{neg}$	$SC^{10^4}_{pos}$	$SC^{10^5}_{neg}$	$SC^{10^5}_{pos}$
25%	41.57%	18.89%	42.3%	26.28%	27.64%	22.71%	39.51%	40.24%
50%	32.22%	12.83%	34.57%	14.55%	12.33%	10.81%	30.49%	30.99%
75%	18.47%	3.85%	24.76%	4.0%	11.76%	6.25%	15.29%	6.42%
**AUC**	$emo-net^a$	$emo-net^b$	$emo-net^c$	$emo-net^d$	$SC^{10^4}_{neg}$	$SC^{10^4}_{pos}$	$SC^{10^5}_{neg}$	$SC^{10^5}_{pos}$
25%	59.75%	45.14%	60.97%	52.17%	54.54%	60.89%	59.73%	68.98%
50%	54.08%	33.77%	52.14%	49.43%	54.12%	52.18%	53.18%	56.11%
75%	56.0%	7.0%	39.9%	35.09%	26.67%	13.33%	45.78%	63.62%

The results in terms of precision, the F1 score and AUC for the weighted Jaccard coefficient for the 25%, 50% and 75% of links in hashtags networks on eight datasets.

Table 14: **PRECISION, F1 score and AUC for the weighted preferential attachment**

**PRECISION**	$emo-net^a$	$emo-net^b$	$emo-net^c$	$emo-net^d$	$SC_{neg}^{10^4}$	$SC_{pos}^{10^4}$	$SC_{neg}^{10^5}$	$SC_{pos}^{10^5}$
25%	32.14%	27.36%	34.35%	34.69%	34.91%	31.53%	37.27%	43.85%
50%	18.43%	19.76%	26.76%	23.77%	20.44%	26.43%	26.83%	30.0%
75%	10.62%	10.78%	15.76%	12.24%	13.75%	12.9%	17.83%	23.76%
**F1**	$emo-net^a$	$emo-net^b$	$emo-net^c$	$emo-net^d$	$SC_{neg}^{10^4}$	$SC_{pos}^{10^4}$	$SC_{neg}^{10^5}$	$SC_{pos}^{10^5}$
25%	48.65%	42.97%	51.14%	51.51%	51.75%	47.94%	54.3%	60.97%
50%	31.13%	33.0%	42.22%	38.41%	33.94%	41.81%	42.31%	46.15%
75%	19.2%	19.47%	27.23%	21.82%	24.18%	22.86%	30.27%	38.39%
**AUC**	$emo-net^a$	$emo-net^b$	$emo-net^c$	$emo-net^d$	$SC_{neg}^{10^4}$	$SC_{pos}^{10^4}$	$SC_{neg}^{10^5}$	$SC_{pos}^{10^5}$
25%	58.45%	62.3%	62.5%	60.92%	75.03%	76.2%	66.81%	69.21%
50%	53.84%	68.63%	59.64%	54.72%	66.48%	69.29%	58.75%	63.67%
75%	64.19%	70.78%	54.32%	36.85%	64.69%	44.1%	62.22%	60.45%

The results in terms of precision, the F1 score and AUC for the weighted preferential attachment for the 25%, 50% and 75% of links in hashtags networks on eight datasets.

Table 15: **PRECISION, F1 score and AUC for the weighted Adamic-Adar**

**PRECISION**	$emo-net^a$	$emo-net^b$	$emo-net^c$	$emo-net^d$	$SC_{neg}^{10^4}$	$SC_{pos}^{10^4}$	$SC_{neg}^{10^5}$	$SC_{pos}^{10^5}$
25%	35.25%	27.76%	38.39%	36.29%	32.55%	31.53%	37.1%	43.1%
50%	24.27%	21.77%	29.1%	26.01%	17.52%	20.71%	24.09%	30.48%
75%	15.04%	12.75%	16.3%	14.29%	15.0%	9.68%	15.92%	22.1%
**F1**	$emo-net^a$	$emo-net^b$	$emo-net^c$	$emo-net^d$	$SC_{neg}^{10^4}$	$SC_{pos}^{10^4}$	$SC_{neg}^{10^5}$	$SC_{pos}^{10^5}$
25%	52.12%	43.45%	55.48%	53.25%	49.11%	47.94%	54.12%	60.23%
50%	39.06%	35.76%	45.08%	41.28%	29.81%	34.32%	38.82%	46.72%
75%	26.15%	22.61%	28.04%	25.0%	26.09%	17.65%	27.47%	36.2%
**AUC**	$emo-net^a$	$emo-net^b$	$emo-net^c$	$emo-net^d$	$SC_{neg}^{10^4}$	$SC_{pos}^{10^4}$	$SC_{neg}^{10^5}$	$SC_{pos}^{10^5}$
25%	60.86%	63.16%	65.73%	62.96%	69.1%	63.64%	64.85%	70.16%
50%	59.01%	57.47%	58.8%	58.06%	67.22%	67.19%	62.89%	60.65%
75%	58.01%	58.08%	49.33%	40.88%	61.83%	51.19%	63.55%	65.11%

The results in terms of precision, the F1 score and AUC for the weighted Adamic-Adar for the 25%, 50% and 75% of links in hashtags networks on eight datasets.

Table 16: **PRECISION, F1 score and AUC for the resource allocation index**

**PRECISION**	$emo-net^a$	$emo-net^b$	$emo-net^c$	$emo-net^d$	$SC^{10^4}_{neg}$	$SC^{10^4}_{pos}$	$SC^{10^5}_{neg}$	$SC^{10^5}_{pos}$
25%	36.34%	22.24%	35.55%	33.21%	29.25%	22.66%	31.87%	36.57%
50%	24.42%	16.53%	25.0%	23.99%	13.87%	12.14%	22.87%	24.05%
75%	18.14%	6.86%	14.13%	14.8%	10.0%	6.45%	12.74%	11.05%
**F1**	$emo-net^a$	$emo-net^b$	$emo-net^c$	$emo-net^d$	$SC^{10^4}_{neg}$	$SC^{10^4}_{pos}$	$SC^{10^5}_{neg}$	$SC^{10^5}_{pos}$
25%	53.3%	36.39%	52.45%	49.86%	45.26%	36.95%	48.34%	53.56%
50%	39.26%	28.37%	40.0%	38.7%	24.36%	21.66%	37.22%	38.77%
75%	30.71%	12.84%	24.76%	25.78%	18.18%	12.12%	22.6%	19.9%
**AUC**	$emo-net^a$	$emo-net^b$	$emo-net^c$	$emo-net^d$	$SC^{10^4}_{neg}$	$SC^{10^4}_{pos}$	$SC^{10^5}_{neg}$	$SC^{10^5}_{pos}$
25%	61.96%	68.0%	58.08%	64.42%	61.55%	56.95%	63.6%	59.18%
50%	58.64%	55.17%	59.87%	62.76%	58.72%	44.4%	61.09%	54.98%
75%	68.03%	50.9%	55.61%	59.33%	53.99%	60.56%	63.28%	56.13%

The results in terms of precision, the F1 score and AUC for the resource allocation index for the 25%, 50% and 75% of links in hashtags networks on eight datasets.

Table 17: **PRECISION, F1 score and AUC for selectivity**

**PRECISION**	$emo-net^a$	$emo-net^b$	$emo-net^c$	$emo-net^d$	$SC^{10^4}_{neg}$	$SC^{10^4}_{pos}$	$SC^{10^5}_{neg}$	$SC^{10^5}_{pos}$
25%	36.02%	22.24%	38.39%	33.46%	26.42%	18.23%	31.03%	31.11%
50%	26.11%	18.95%	31.64%	24.66%	11.68%	15.0%	20.73%	20.48%
75%	16.81%	13.73%	20.65%	17.86%	6.25%	8.06%	12.74%	7.73%
**F1**	$emo-net^a$	$emo-net^b$	$emo-net^c$	$emo-net^d$	$SC^{10^4}_{neg}$	$SC^{10^4}_{pos}$	$SC^{10^5}_{neg}$	$SC^{10^5}_{pos}$
25%	52.97%	36.39%	55.48%	50.14%	41.79%	30.83%	47.36%	47.45%
50%	41.41%	31.86%	48.07%	39.57%	20.92%	26.09%	34.34%	33.99%
75%	28.79%	24.14%	34.23%	30.3%	11.76%	14.93%	22.6%	14.36%
**AUC**	$emo-net^a$	$emo-net^b$	$emo-net^c$	$emo-net^d$	$SC^{10^4}_{neg}$	$SC^{10^4}_{pos}$	$SC^{10^5}_{neg}$	$SC^{10^5}_{pos}$
25%	66.96%	70.16%	71.41%	69.13%	65.73%	74.08%	60.94%	67.2%
50%	60.26%	61.33%	68.01%	58.1%	53.85%	55.24%	61.27%	58.07%
75%	63.05%	59.9%	60.96%	67.33%	38.53%	48.6%	51.13%	56.8%

The results in terms of precision, the F1 score and AUC for selectivity for the 25%, 50% and 75% of links in hashtags networks on eight datasets.

Table 18: **PRECISION, F1 score and AUC for inverse selectivity**

**PRECISION**	$emo-net^a$	$emo-net^b$	$emo-net^c$	$emo-net^d$	$SC^{10^4}_{neg}$	$SC^{10^4}_{pos}$	$SC^{10^5}_{neg}$	$SC^{10^5}_{pos}$
25%	38.9%	23.23%	38.5%	34.93%	29.72%	23.15%	33.05%	37.33%
50%	27.8%	22.58%	30.86%	29.37%	13.87%	16.43%	23.17%	24.05%
75%	18.58%	16.67%	21.2%	20.41%	8.75%	14.52%	14.01%	13.81%
**F1**	$emo-net^a$	$emo-net^b$	$emo-net^c$	$emo-net^d$	$SC^{10^4}_{neg}$	$SC^{10^4}_{pos}$	$SC^{10^5}_{neg}$	$SC^{10^5}_{pos}$
25%	56.01%	37.7%	55.59%	51.78%	45.82%	37.6%	49.68%	54.36%
50%	43.51%	36.84%	47.16%	45.41%	24.36%	28.22%	37.62%	38.77%
75%	31.34%	28.57%	34.98%	33.9%	16.09%	25.35%	24.58%	24.27%
**AUC**	$emo-net^a$	$emo-net^b$	$emo-net^c$	$emo-net^d$	$SC^{10^4}_{neg}$	$SC^{10^4}_{pos}$	$SC^{10^5}_{neg}$	$SC^{10^5}_{pos}$
25%	65.53%	70.73%	65.24%	68.93%	67.48%	65.68%	64.13%	65.16%
50%	61.13%	64.17%	64.2%	61.19%	65.1%	60.85%	63.09%	60.52%
75%	67.77%	52.56%	60.73%	60.62%	68.88%	46.12%	70.17%	50.31%

The results in terms of precision, the F1 score and AUC for inverse selectivity for the 25%, 50% and 75% of links in hashtags networks on eight datasets.

3.5 Results for the networks with the top 200 and the top 500 hashtags in $SC_{pos}^{10^5}$

In this section we list results for the 25%, 50% and 75% networks of hastags in $SC_{pos}^{10^5}$ for **the top 200 and the top 500 hashtags**. The results are reported for precision, the F1 score and the AUC for each of the tested link prediction measures: the weighted common neighbors in Table 19, the weighted Jaccard coefficient in Table 20, the weighted preferential attachment in Table 21, the weighted Adamic-Adar in Table 22, the resource allocation index in Table 23, selectivity in Table 24 and inverse selectivity in Table 25.

Table 19: **PRECISION, F1 score and AUC for the weighted common neighbors**

**PRECISION**	$SC_{pos}^{10^5}$ (top 200)	$SC_{pos}^{10^5}$ (top 500)
25%	42.64%	35.78%
50%	30.0%	22.05%
75%	20.44%	14.3%
**F1**	$SC_{pos}^{10^5}$ (top 200)	$SC_{pos}^{10^5}$ (top 500)
25%	59.79%	52.7%
50%	46.15%	36.13%
75%	33.94%	25.03%
**AUC**	$SC_{pos}^{10^5}$ (top 200)	$SC_{pos}^{10^5}$ (top 500)
25%	70.19%	59.99%
50%	61.24%	60.6%
75%	70.17%	60.46%

The results in terms of precision, the F1 score and AUC for the weighted common neighbors for the 25%, 50% and 75% of links in the top 200 and the top 500 hashtags networks on $SC_{pos}^{10^5}$ dataset.

Table 20: **PRECISION, F1 score and AUC for the weighted Jaccard coefficient**

**PRECISION**	$SC_{pos}^{10^5}$ (top 200)	$SC_{pos}^{10^5}$ (top 500)
25%	25.19%	24.36%
50%	18.33%	19.32%
75%	3.31%	8.64%
**F1**	$SC_{pos}^{10^5}$ (top 200)	$SC_{pos}^{10^5}$ (top 500)
25%	40.24%	39.18%
50%	30.99%	32.38%
75%	6.42%	15.9%
**AUC**	$SC_{pos}^{10^5}$ (top 200)	$SC_{pos}^{10^5}$ (top 500)
25%	68.98%	62.72%
50%	56.11%	51.87%
75%	63.62%	45.98%

The results in terms of precision, the F1 score and AUC for the weighted Jaccard coefficient for the 25%, 50% and 75% of links in the top 200 and the top 500 hashtags networks on $SC_{pos}^{10^5}$ dataset.

Table 21: **PRECISION, F1 score and AUC for the weighted preferential attachment**

**PRECISION**	$SC_{pos}^{10^5}$ (top 200)	$SC_{pos}^{10^5}$ (top 500)
25%	43.85%	45.49%
50%	30.0%	33.52%
75%	23.76%	23.75%
**F1**	$SC_{pos}^{10^5}$ (top 200)	$SC_{pos}^{10^5}$ (top 500)
25%	60.97%	62.53%
50%	46.15%	50.21%
75%	38.39%	38.39%
**AUC**	$SC_{pos}^{10^5}$ (top 200)	$SC_{pos}^{10^5}$ (top 500)
25%	69.21%	70.93%
50%	63.67%	65.85%
75%	60.45%	61.48%

The results in terms of precision, the F1 score and AUC for the weighted preferential attachment for the 25%, 50% and 75% of links in the top 200 and the top 500 hashtags networks on $SC_{pos}^{10^5}$ dataset.

Table 22: **PRECISION, F1 score and AUC for the weighted Adamic-Adar**

**PRECISION**	$SC_{pos}^{10^5}$ (top 200)	$SC_{pos}^{10^5}$ (top 500)
25%	43.1%	36.8%
50%	30.48%	23.05%
75%	22.1%	15.38%
**F1**	$SC_{pos}^{10^5}$ (top 200)	$SC_{pos}^{10^5}$ (top 500)
25%	60.23%	53.8%
50%	46.72%	37.47%
75%	36.2%	26.67%
**AUC**	$SC_{pos}^{10^5}$ (top 200)	$SC_{pos}^{10^5}$ (top 500)
25%	70.16%	60.63%
50%	60.65%	61.29%
75%	65.11%	62.74%

The results in terms of precision, the F1 score and AUC for the weighted Adamic-Adar for the 25%, 50% and 75% of links in the top 200 and the top 500 hashtags networks on $SC_{pos}^{10^5}$ dataset.

Table 23: **PRECISION, F1 score and AUC for the resource allocation index**

**PRECISION**	$SC_{pos}^{10^5}$ (top 200)	$SC_{pos}^{10^5}$ (top 500)
25%	36.57%	35.88%
50%	24.05%	26.22%
75%	11.05%	14.71%
**F1**	$SC_{pos}^{10^5}$ (top 200)	$SC_{pos}^{10^5}$ (top 500)
25%	53.56%	52.81%
50%	38.77%	41.55%
75%	19.9%	25.65%
**AUC**	$SC_{pos}^{10^5}$ (top 200)	$SC_{pos}^{10^5}$ (top 500)
25%	59.18%	64.89%
50%	54.98%	59.72%
75%	56.13%	60.32%

The results in terms of precision, the F1 score and AUC for the resource allocation index for the 25%, 50% and 75% of links in the top 200 and the top 500 hashtags networks on $SC_{pos}^{10^5}$ dataset.

Table 24: **PRECISION, F1 score and AUC for selectivity**

**PRECISION**	$SC_{pos}^{10^5}$ (top 200)	$SC_{pos}^{10^5}$ (top 500)
25%	31.11%	35.64%
50%	20.48%	23.05%
75%	7.73%	13.23%
**F1**	$SC_{pos}^{10^5}$ (top 200)	$SC_{pos}^{10^5}$ (top 500)
25%	47.45%	52.55%
50%	33.99%	37.47%
75%	14.36%	23.36%
**AUC**	$SC_{pos}^{10^5}$ (top 200)	$SC_{pos}^{10^5}$ (top 500)
25%	67.2%	66.94%
50%	58.07%	65.3%
75%	56.8%	63.76%

The results in terms of precision, the F1 score and AUC for selectivity for the 25%, 50% and 75% of links in the top 200 and the top 500 hashtags networks on $SC_{pos}^{10^5}$ dataset.

Table 25: **PRECISION, F1 score and AUC for inverse selectivity**

**PRECISION**	$SC_{pos}^{10^5}$ (top 200)	$SC_{pos}^{10^5}$ (top 500)
25%	37.33%	38.74%
50%	24.05%	27.84%
75%	13.81%	16.6%
**F1**	$SC_{pos}^{10^5}$ (top 200)	$SC_{pos}^{10^5}$ (top 500)
25%	54.36%	55.84%
50%	38.77%	43.55%
75%	24.27%	28.47%
**AUC**	$SC_{pos}^{10^5}$ (top 200)	$SC_{pos}^{10^5}$ (top 500)
25%	65.16%	68.58%
50%	60.52%	64.63%
75%	50.31%	60.85%

The results in terms of precision, the F1 score and AUC for inverse selectivity for the 25%, 50% and 75% of links in the top 200 and the top 500 hashtags networks on $SC_{pos}^{10^5}$ dataset.
